# Latest Advancements and Mechanistic Insights into High-Entropy Alloys: Design, Properties and Applications

**DOI:** 10.3390/ma18245616

**Published:** 2025-12-14

**Authors:** Anthoula Poulia, Alexander E. Karantzalis

**Affiliations:** Department of Materials Science and Engineering, University of Ioannina, 45110 Ioannina, Greece; akarantz@uoi.gr

**Keywords:** high-entropy alloys (HEAs), materials design, additive manufacturing, machine learning, advanced properties, microstructural evolution, future applications

## Abstract

High-entropy alloys (HEAs) are a class of multi-principal element materials composed of five or more elements in near-equimolar ratios. This unique compositional design generates high configurational entropy, which stabilizes simple solid solution phases and reduces the tendency for intermetallic compound formation. Unlike conventional alloys, HEAs exhibit a combination of properties that are often mutually exclusive, such as high strength and ductility, excellent thermal stability, superior corrosion and oxidation resistance. The exceptional mechanical performance of HEAs is attributed to mechanisms including lattice distortion strengthening, sluggish diffusion, and multiple active deformation pathways such as dislocation slip, twinning, and phase transformation. Advanced characterization techniques such as transmission electron microscopy (TEM), atom probe tomography (APT), and in situ mechanical testing have revealed the complex interplay between microstructure and properties. Computational approaches, including CALPHAD modeling, density functional theory (DFT), and machine learning, have significantly accelerated HEA design, allowing prediction of phase stability, mechanical behavior, and environmental resistance. Representative examples include the FCC-structured CoCrFeMnNi alloy, known for its exceptional cryogenic toughness, Al-containing dual-phase HEAs, such as AlCoCrFeNi, which exhibit high hardness and moderate ductility and refractory HEAs, such as NbMoTaW, which maintain ultra-high strength at temperatures above 1200 °C. Despite these advances, challenges remain in controlling microstructural homogeneity, understanding long-term environmental stability, and developing cost-effective manufacturing routes. This review provides a comprehensive and analytical study of recent progress in HEA research (focusing on literature from 2022–2025), covering thermodynamic fundamentals, design strategies, processing techniques, mechanical and chemical properties, and emerging applications, through highlighting opportunities and directions for future research. In summary, the review’s unique contribution lies in offering an up-to-date, mechanistically grounded, and computationally informed study on the HEAs research-linking composition, processing, structure, and properties to guide the next phase of alloy design and application.

## 1. Introduction

**Figure 1:**
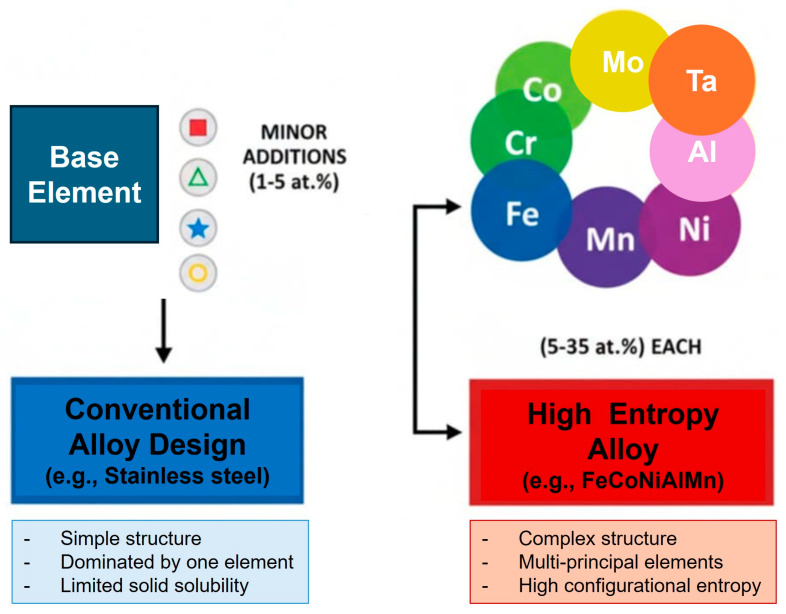
Conceptual schematic illustrating the difference between conventional alloy design (base element with minor additions) versus high-entropy alloy design (multiple principal elements in near-equimolar ratios (≈15–25 at.%)).

The concept of high-entropy alloys (HEAs) has become one of the most transformative developments in materials science, through redefining the traditional alloy design strategies. Unlike conventional alloys, which typically rely on one dominant base element with minor alloying additions, HEAs are composed of multiple principal elements-usually five or more-in near-equiatomic ratios (Figure 1).

The compositional design space of high-entropy alloys (HEAs) is astronomically large and intrinsically high-dimensional. Even restricting to the commonly studied equimolar quinary subspace yields on the order of 10^5^–10^6^ distinct compositions and—when explored with high-throughput first-principles screening—over 6.5 × 10^5^ equimolar quinary alloys have been explicitly examined, from which >3.0 × 10^4^ candidate single-phase solid solutions were predicted (≈5% of the enumerated quinary set), highlighting both the sparsity of stable single-phase regions and the enormous remaining search space [1]. Generally, allowing non-equimolar chemistries and higher-order (k > 5) combinations convert the problem from a large discrete combinatorics task into an effectively continuous, multi-parametric design manifold, so that practical discrete samples rapidly escalate into millions-to-billions of points and brute-force experimental exploration becomes impossible without algorithmic priors.

This compositional complexity results in high configurational entropy, which stabilizes simple solid-solution phases, such as face-centered cubic (FCC), body-centered cubic (BCC), or dual-phase structures, while suppressing the formation of brittle intermetallics [2,3]. Beyond entropy, the interplay of lattice distortion, atomic size mismatch, and valence electron concentration drives unique phase stability and mechanical responses that cannot be achieved in conventional alloys [4].

Recent studies highlight the versatility of HEAs in achieving outstanding property combinations across different alloy systems. For example, the prototypical CoCrFeMnNi alloy retains a single-phase FCC structure, exhibiting exceptional ductility (>60% elongation) and cryogenic toughness, attributed to mechanisms such as twinning-induced plasticity (TWIP) and nanoscale stacking fault formation [5,6]. In contrast, Al-containing HEAs, such as AlCuCrFeNi, display dual-phase FCC–BCC microstructures that enhance strength and hardness at the expense of ductility [7]. Refractory HEAs, such as NbMoTaW or HfNbTiZr, are now recognized for maintaining high yield strengths above 1 GPa at temperatures exceeding 1200 °C, largely due to sluggish diffusion and solid-solution strengthening [8,9].

Key parameters, like diffusion coefficients and stacking fault energies (SFE) have also been measured, giving promising results for future HEAs exploration. For instance, in FCC CoCrFeMnNi alloy the measured volume/tracer diffusion coefficients at high temperatures (≈1373 K) lie at D = 1 × 10^−15^–1 × 10^−14^ m^2^·s^−1^ [10]. For BCC HEAs (e.g., AlCoCrCuFeNi), D values are 10^−13^ m^2^/s at 1200 K—roughly 10× faster [11]. In terms of the SFE, FCC system CoCrFeMnNi presents a SFE range of 18–35 mJ·m^−2^, FeCoNiCr alloy exhibits a SFE range of 25–40 mJ·m^−2^, while FeMnCoCr system has a SFE range of 10–30 mJ·m^−2^. FCC/BCC system Al_x_CoCrFeNi_x_ has a SFE range of 40–120 mJ·m^−2^ [12].

Quantifying short-range order (SRO) in HEAs is crucial for understanding their atomic-scale structure and how it affects mechanical, thermal, and magnetic properties. The most widely used quantitative measures are based on pair correlation statistics, with the Warren–Cowley short-range order (SRO) parameter being the canonical descriptor. For example, in the well-studied Cantor alloys CoCrFeNiMn, Co-Cr pair shows a weak preference for mixing (a_1_ = −0.05 and a_2_ = +0.02) and Fe-Ni pair shows a slight tendency for clustering (a_1_ = +0.04 and a_2_ = −0.01).

The rapid evolution of HEA research has also been fueled by computational modeling and data-driven methods. CALPHAD calculations, density functional theory (DFT), and molecular dynamics are routinely used to predict phase stability and defect interactions [13]. More recently, machine learning and artificial intelligence have been integrated with experimental databases to accelerate HEAs discovery, enabling prediction of unexplored compositions with tailored properties [14,15]. Such approaches are especially powerful, given the vast compositional design space of HEAs, which makes experimental trial-and-error prohibitively expensive and time-consuming.

Advanced manufacturing routes are further expanding HEA applications. Additive manufacturing (AM)—and especially selective laser melting and electron beam melting—has enabled the production of near-dense HEA components with refined microstructures, enhanced strength, and site-specific compositional tuning [16,17]. For instance, AlCoCrFeNi-based alloys processed via AM have achieved compressive strengths exceeding 2 GPa, while maintaining useful ductility [18]. Similarly, powder metallurgy and mechanical alloying approaches have demonstrated improved microstructural uniformity and oxidation resistance in both lightweight and refractory HEAs [19].

Beyond mechanical properties, functional HEAs have emerged for applications in energy storage, catalysis [20], and corrosion resistance. Multi-principal element compositions allow tuning of surface electronic structures for enhanced electrocatalytic activity in hydrogen evolution, oxygen reduction, and CO_2_ reduction reactions [21,22]. Latest innovations in surface engineering and nanostructuring of HEAs have further improved their catalytic efficiency and long-term stability [23]. Likewise, lightweight HEAs with tailored Al, Ti, and Mg additions are being explored for aerospace applications, where high specific strength and oxidation resistance are critical [24].

Despite remarkable progress, several challenges hinder the widespread adoption of HEAs. High production costs, compositional inhomogeneities during large-scale processing, and insufficient understanding of long-term stability under service conditions remain critical barriers [25]. Emerging strategies such as combinatorial high-throughput synthesis, machine learning-guided screening, and multi-scale modeling are being actively pursued to address these challenges [26]. Continued integration of computational predictions with experimental validation is expected to pave the way for the rational design and industrial deployment of HEAs across structural, high-temperature, and functional domains [27].

## 2. Thermodynamic and Structural Fundamentals

HEAs have emerged as a transformative class of materials due to their multi-principal element compositions, typically comprising five or more elements in near-equiatomic ratios. This compositional complexity gives rise to a unique interplay of thermodynamic, kinetic, and structural factors that dictate phase formation, microstructural evolution, and mechanical performance. In the last three years, substantial progress has been made in understanding these fundamentals, providing new insights into the design and optimization of HEAs for structural, functional, and high-temperature applications.

### 2.1. Classical Thermodynamic Descriptors—Definitions and Typical Thresholds

**Figure 2:**
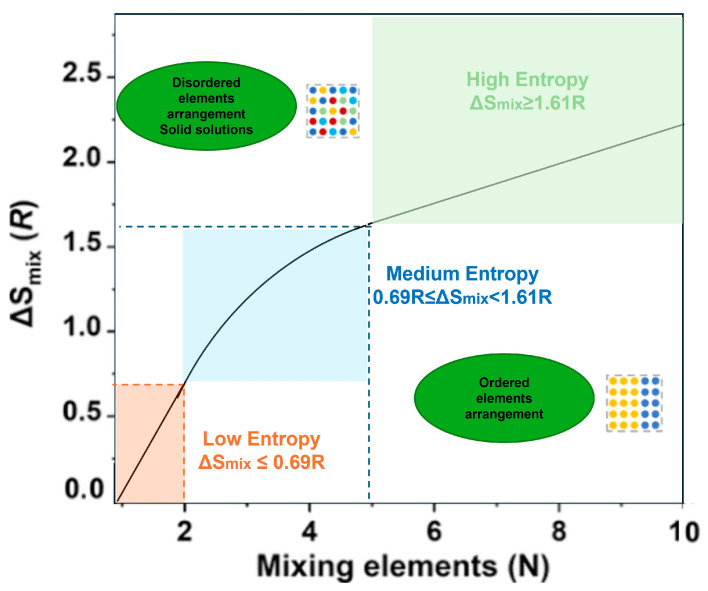
Plot of configurational entropy versus number of principal elements, illustrating stabilization of HEAs.

The thermodynamic stability of HEAs is largely governed by the high configurational entropy of mixing, which is maximized when multiple elements are present in nearly equimolar ratios. For a system containing N components, the configurational entropy is defined as ΔS_conf_ = RlnN, where R is the gas constant. For a five-component HEA, this yields a configurational entropy of approximately 13.38 J/mol·K (Figure 2). Such high entropy can substantially reduce the Gibbs free energy of a disordered solid solution relative to competing ordered intermetallics, thereby favoring simple face-centered cubic (FCC) or body-centered cubic (BCC) lattices. However, recent studies emphasize that configurational entropy alone is not sufficient to fully predict phase stability. Vibrational, electronic, and magnetic contributions to entropy, as well as enthalpic interactions between constituent elements, must also be accounted for to accurately describe phase equilibria.

Specifically, the total free energy F_total_(T,V) is expressed as: F_total_(T,V) = E_elec_(V) + F_vib_(T,V) + F_mag_(T,V) − TS_conf_(T), where:

- E_elec_(V) is the ground-state electronic energy calculated from density functional theory (DFT). It is computed at different atomic configurations and volumes to establish the static lattice energy curve [28].
- F_vib_(T,V) is the vibrational free energy, which can be obtained using the quasiharmonic approximation (QHA): F_vib_(T,V) = k_B_T∫g(ω,V)ln [2sinh(ℏω/2k_B_T)]dω, where g(ω,V) is the phonon density of states [29]. Thermal expansion effects were considered within the quasiharmonic approximation by allowing the equilibrium volume V(T) to minimize F_total_(T,V) at each temperature: (∂F_total_(T,V)/∂V)_T_ = 0. This approach inherently accounts for isotropic thermal expansion, which is a reasonable assumption for cubic HEAs. For systems exhibiting significant anisotropy, an anisotropic expansion model can be introduced through independent variation in lattice parameters a, b, c [30].
- F_mag_(T,V) represents the magnetic contribution, often described by the disordered local moment (DLM) model [31] or mean-field approximation [32]. F_mag_(T,V) captures the magnetic disorder at finite temperatures [33]: F_mag_(T,V) = −k_B_Tln[(sinh(μ_B_/k_B_T))/(μ_B_/k_B_T)], where μ is the magnetic moment and B is the effective magnetic field.
- S_conf_ = −k_B_∑c_i_lnc_i_ is the configurational entropy, where c_i_ represents the atomic fraction of each constituent element.

Calorimetric measurements on CoCrFeMnNi alloys have shown that the configurational contribution to the Gibbs free energy accounts for roughly 25% of the total stabilization, while vibrational and magnetic contributions further stabilize the FCC phase at high temperatures [3,26].

In more details, the “total stabilization” term refers to the overall reduction in Gibbs free energy (ΔG_(stabilization)_ = G_(competing)_ − G₍_FCC_₎) that favors the disordered solid solution, while the configurational contribution represents only the entropy-driven part of this stabilization. For an equimolar five-component alloy, the ideal configurational entropy (S₍_config_₎ = R ln 5 ≈ 13.4 J mol^−1^ K^−1^) corresponds to a free-energy term of approximately −13 kJ mol^−1^ at 1000 K. When compared with the typical total stabilization of 40–50 kJ mol^−1^, this configurational term contributes about 25–30% of the total Gibbs free-energy reduction. The remaining stabilization arises from non-configurational factors such as mixing enthalpy, local lattice distortion and relaxation effects, vibrational and magnetic entropies, as well as electronic contributions, which together determine the precise energetic balance between FCC, BCC, and ordered intermetallic structures. Therefore, while the high configurational entropy characteristic of multi-principal-element systems provides a significant thermodynamic driving force for maintaining a single-phase solid solution-particularly at elevated temperatures-it is not the sole factor controlling structural stability. Enthalpic interactions and other entropic terms often dominate at lower temperatures or under specific compositional and processing conditions. This interpretation aligns with thermodynamic analyses and first-principles calculations reported in the literature, which demonstrate that configurational entropy alone cannot fully account for phase stabilization in HEAs but operates synergistically with other energetic contributions [34,35,36,37,38].

While high entropy can favor disordered solid solutions, the enthalpy of mixing (ΔH_mix_) exerts a competing influence. Small negative or near-zero mixing enthalpies are generally conducive to the formation of single-phase solid solutions, whereas highly negative values often lead to ordering or the formation of intermetallic compounds. Recent meta-analyses of FCC HEAs indicate that compositions with −10 kJ/mol < ΔH_mix_ < 5 kJ/mol typically result in single-phase FCC structures, whereas refractory BCC HEAs can tolerate slightly more negative mixing enthalpies, up to −15 kJ/mol, before phase separation occurs [39,40].

### 2.2. Refined Descriptors for HEAs Modeling

**Figure 3:**
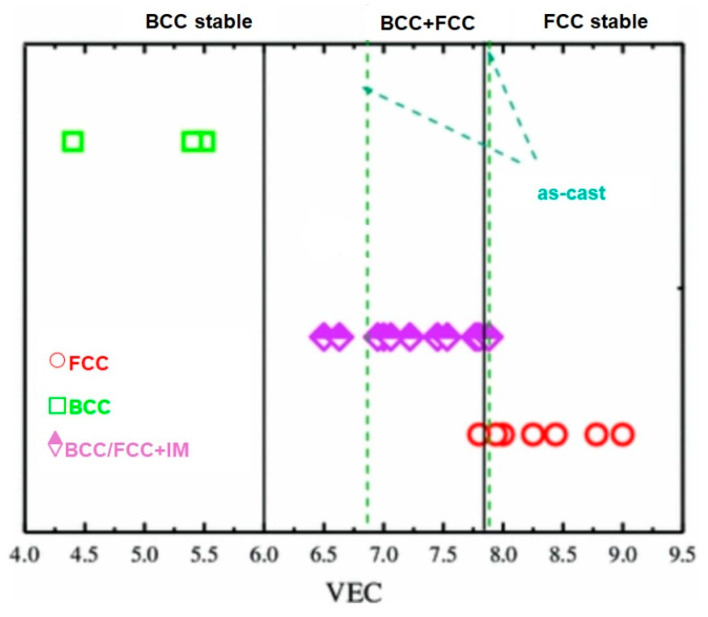
Schematic showing phase stability regions (FCC, BCC, dual-phase) as a function of valence electron concentration (VEC).

As research on HEAs progressed, limitations of the classical descriptors became clear. New parametric models refer to descriptor sets that go beyond simple averages and incorporate local environment, structural-distortion, electronic features, etc.:

- Atomic size mismatch, represented by the parameter δ, plays a crucial role in stabilizing solid solutions and controlling lattice distortion. The parameter is defined as δ = sqrt[(∑i*c_i_) ∗ (1 − r_i_/r_m_)^2^)] × 100%, where c_i_ and r_i_ are the atomic fraction and atomic radius of element i. FCC HEAs typically exhibit a lattice mismatch of 3–6%, while refractory BCC HEAs can reach 8–12%. These distortions contribute to solid-solution strengthening by impeding dislocation motion and altering defect energetics, and recent high-resolution studies using synchrotron XRD and EXAFS have quantified these effects across multiple HEA systems [41,42].
- Valence electron concentration (VEC) remains a key electronic parameter that correlates strongly with the preferred crystal structure. Compositions with VEC greater than 8, tend to stabilize FCC structures, whereas VEC below 6.8, favors BCC lattices. Alloys with intermediate VEC values often result in mixed-phase microstructures (Figure 3). Recent investigations have refined these thresholds through systematic experimentation and DFT-based electronic structure calculations, demonstrating that subtle changes in composition can shift the dominant lattice type and profoundly influence mechanical properties such as stacking fault energy (SFE) and ductility [9].

A further deepening in the phase formation prediction models proposes additional or modified descriptors to capture geometric/electronic effects.

- Φ (entropy/entropy-excess) parameter. Dimensionless Φ parameter has been proposed to delineate single-, two- and multiphase regions, with suggested empirical thresholds (Φ_c_ ≈ 20 separating single vs. multiphase behavior in some studies). This parameter attempts to combine entropy and size mismatch in ways that collapse larger data sets [43].
- Electronegativity-based metrics and Δ_χ_ (average electronegativity difference). Simple electronegativity spread or variance (e.g., Δ_χ_ = ∑_i_c_i_(χ_i_ − χ^−^)^2^ has been used to penalize compositions with large chemical driving forces for ordering/compound formation [44]. More recent works augment Δ_χ_ with local environment weighing or combine it with valence/d-band occupancy concepts to capture charge transfer and electronic structure effects. These electronegativity-type modifiers improve discrimination in some alloy classes (especially when strong compound-forming pairs exist) but are not a universal fix [45,46].

### 2.3. Critical Comparison Between the Classical Rules vs. Computational and Experimental Suggestions

The thermodynamic criteria for phase prediction remain useful as a quick filter and design heuristic. However, broad surveys and targeted experimental tests in 2023–2025 show numerous counterexamples: alloys that satisfy these heuristic thresholds yet form intermetallics or phase separation tendency under realistic processing. Additionally, alloys that violate one or more thresholds yet produce single-phase solid solutions because of kinetic trapping, local ordering, or peculiar binary interaction energetics [47,48,49].

Thus, the vast compositional space of HEAs necessitates more sophisticated thermodynamic modeling beyond simple empirical rules. Modern progress has integrated CALPHAD-based phase diagram calculations with machine learning approaches to predict phase stability, order–disorder transitions, and the formation of metastable phases. These hybrid methods allow rapid exploration of thousands of potential compositions, providing probabilistic predictions of phase formation and enabling more efficient design strategies. For example, ML-assisted CALPHAD models have successfully predicted the phase behavior of Al_x_CoCrFeNi alloys, accurately capturing the transition from single-phase FCC to dual-phase FCC + BCC structures with increasing Al content [50,51].

Modern DFT studies also provide two important refinements:

1. Binary interaction and formation energies matter. First-principles formation energies for binary pairs (and short-range order tendencies) can be assembled into mixture models; these energies frequently explain why an alloy that looks “entropy-favored” by Ω parameter, still decomposes—because binary pairs have strongly negative formation enthalpies that drive ordering. Recent work explicitly builds classifiers from DFT-derived pairwise interaction features and demonstrates accuracy comparable to or better than models based purely on empirical scalar descriptors, while also offering mechanistic interpretability (which binaries dominate the tendency to order). This work shows DFT-derived interaction features materially improve predictive power [52].
2. Electronic structure and local environment rules. DFT reveals that local charge transfer and the d-band occupancy of specific sites control cohesive energy changes and ordering tendencies; descriptors that encode d-band filling or local electronegativity environment improve discrimination of phases (especially for transition-metal-rich HEAs and catalytic HEAs). New studies propose linear combinations of d-band filling + neighborhood electronegativity as robust predictors for both catalytic activity and phase stability. These electronic descriptors partially subsume what the simple Δχ tries to capture, but with stronger physical grounding [53].

Kinetics and diffusion in HEAs also remain an area of active investigation, particularly in the context of the so-called “sluggish diffusion” hypothesis. Initially proposed to explain enhanced high-temperature stability and creep resistance, this hypothesis suggested that atomic mobility in HEAs is intrinsically slower due to complex local environments and energy barriers. However, recent tracer diffusion studies and molecular dynamics simulations have revealed a more nuanced picture. For FCC CoCrFeMnNi, tracer diffusion coefficients at 1000 K are comparable to those of conventional Ni-based alloys, whereas refractory BCC HEAs such as NbMoTaW exhibit significantly reduced diffusion, consistent with large lattice distortions and site-specific activation barriers [5,54]. Moreover, short-range chemical ordering has been shown to modulate diffusion pathways, either accelerating or impeding atomic transport depending on local bonding environments. These findings indicate that diffusion in HEAs is highly composition- and site-dependent, and that the concept of universally “sluggish” diffusion should be replaced with a more contextual understanding [55].

Lattice distortion is another defining structural characteristic of HEAs. Severe local strain fields arise from atomic size differences, affecting dislocation behavior, work hardening, and stacking fault energies. Recent high-resolution studies have quantified bond-length variations in FCC and BCC HEAs, with deviations ranging from ±2–4% in FCC alloys to ±5–8% in refractory BCC systems [56]. Such lattice distortions are directly linked to enhanced strength and ductility, as they modify the energy barriers for dislocation motion and the formation of twins or stacking faults. In addition, short-range ordering (SRO) has emerged as a key factor influencing mechanical behavior and phase evolution. For instance, CoCrNi HEAs with SRO exhibit enhanced work-hardening rates due to stabilized nanoscale stacking faults, while in refractory alloys, controlled SRO suppresses the nucleation of deleterious topologically close-packed (TCP) phases [57].

The structural outcomes of HEAs can vary widely depending on composition, processing, and temperature. Single-phase FCC HEAs, such as CoCrFeMnNi, are notable for their combination of high ductility and moderate strength. Alloying with interstitial elements such as carbon or nitrogen can tune the stacking fault energy, promoting deformation mechanisms like twinning-induced plasticity (TWIP). For example, the addition of 0.5 at.% carbon reduces the SFE of CoCrFeMnNi from 25 mJ/m^2^ to approximately 15 mJ/m^2^, enhancing strain hardening and toughness [58]. In contrast, Al-rich HEAs such as AlCoCrFeNi typically form dual-phase FCC + BCC microstructures with higher hardness and yield strength, although with a modest reduction in ductility. Refractory BCC HEAs containing Nb, Mo, Ta, and W can form complex microstructures with B2 or Laves phase precipitates, providing exceptional strength at temperatures exceeding 1200 °C [59]. Under rapid solidification conditions or additive manufacturing, HEAs can form nanocrystalline or amorphous phases with enhanced surface hardness, corrosion resistance, and catalytic activity, expanding their potential applications [60].

### 2.4. Ongoing Debates, Outstanding Challenges and Future Directions of HEAs Designing Models

The thermodynamic and structural foundations of HEAs continue to pose deep, interlinked challenges: classical descriptors (configurational entropy, atomic size mismatch, electronegativity difference) capture only part of the stabilization picture and fail to predict why vibrational and electronic entropic contributions, as well as short-range chemical order, often dominate phase selection in many multi-principal-element systems [61].

The field of HEAs is converging away from its original, sometimes-sweeping claims toward a more nuanced debate focused on which of the purported “core effects” are universal, which are composition- and processing-dependent, and how to exploit (or avoid) them in design. The idea that configurational entropy alone stabilizes single-phase solid solutions is now treated cautiously, with thermodynamic modeling and experiments showing that enthalpic interactions, kinetics and local chemistry often dominate phase selection [61].

Sluggish diffusion, once proposed as a general kinetic advantage of HEAs, is likewise contested—many studies report composition- and temperature-dependent diffusion behavior and rapid precipitation in some alloys, so sluggishness cannot be invoked as a universal explanation for high-temperature stability [62].

A second, active debate concerns short-range order (SRO) and local chemical clustering. Atomistic simulations and recent experiments link SRO to modified stacking-fault energies, dislocation pinning and altered creep pathways in refractory and other HEAs, yet SRO’s net effect on long-term creep, phase separation, and cavity formation appears strongly coupled to grain size, vacancy kinetics and heat-treatment history, making its role context-sensitive rather than categorically beneficial or harmful [63].

Allied controversies address whether the so-called “cocktail” or synergistic effects are real emergent phenomena or simply the sum of many element-specific contributions (and thus predictable by physics-based models), and whether machine-learning/computational screening can reliably find robust compositions given the immense compositional space and sensitive processing dependence [64].

Current CALPHAD and mean-field models struggle to represent these non-ideal entropic and enthalpic terms quantitatively across wide compositional spaces, because available binary/ternary reference data are sparse and first-principles treatments of vibrational and magnetic entropy remain computationally expensive and incomplete for multi-component alloys [65].

On the structural side, severe local lattice distortions, chemical short-range order and heterogeneous strain fields—which decisively control mechanical behavior, defect energetics and phase transformations—are poorly captured by continuum or average-structure descriptions, requiring atomistic descriptors beyond average lattice constants [66].

Kinetics and non-equilibrium pathways (rapid solidification, additive manufacturing, severe plastic deformation) further decouple observed microstructures from equilibrium thermodynamic predictions, exposing a need for coupled thermodynamic-kinetic frameworks that include sluggish diffusion, interface energetics and transient segregation [67].

Moving forward, the most promising directions combine targeted expansion of high-quality thermochemical databases, systematic calorimetry and in situ structural probes with integrated multiscale modeling and machine-learning accelerants that can infer effective entropic/enthalpic descriptors and uncertainty bounds—enabling anticipatory design rules that link composition, local structure and processing to property envelopes [68].

## 3. Computational Approaches

**Figure 4:**
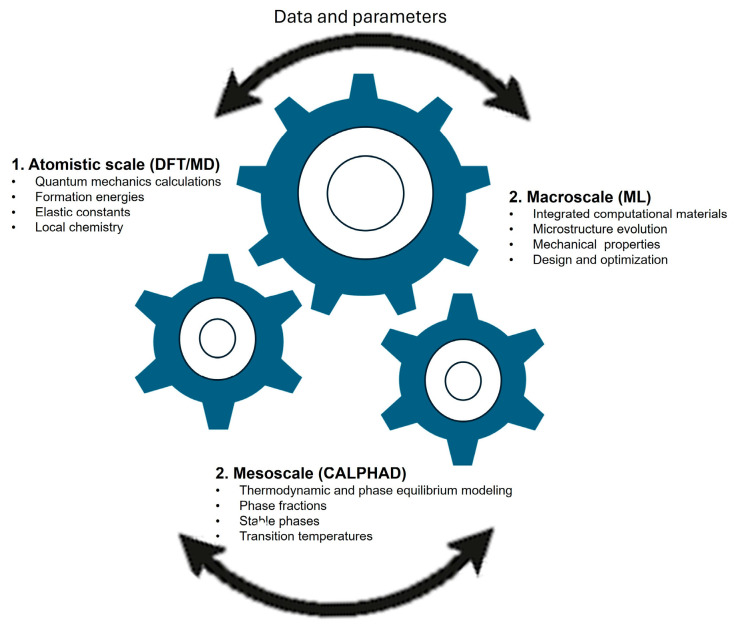
Multiscale computational framework for HEA design: from DFT, CALPHAD and ML models to experimental validation.

The unprecedented compositional complexity of HEAs makes computational modeling indispensable for predicting phase stability, guiding alloy discovery, and understanding fundamental mechanisms across multiple length scales. Over the past decade, computational approaches have evolved from conventional thermodynamic modeling into sophisticated, multiscale frameworks that couple first-principles simulations, atomistic methods, and machine learning (ML) (Figure 4). These tools collectively accelerate the exploration of compositional spaces that are practically inaccessible to exhaustive experimental study [69,70,71,72,73].

### 3.1. First-Principles Density Functional Theory (DFT) of HEAs

First-principles density functional theory (DFT) remains the cornerstone for evaluating fundamental properties such as mixing enthalpies, elastic constants, stacking-fault energies, and defect energetics [74,75,76]. High-throughput frameworks have been implemented to screen thousands of multicomponent compositions by constructing representative models such as special quasi-random structures (SQS) [77].

SQS are computational models designed to approximate the atomic arrangement of random solid solutions within a finite periodic supercell. By constructing a supercell whose short-range correlation functions (such as pair and multi-site correlations) closely reproduce those of a perfectly random alloy, the SQS method enables efficient simulation of chemical disorder using first-principles calculations, such as DFT, without the need to model a vast number of configurations. This approach, first introduced by Zunger et al. [78], has become a powerful tool for studying multicomponent systems, particularly HEAs, where atomic disorder plays a central role in determining material properties. The SQS framework allows researchers to investigate the thermodynamic stability, electronic structure, and mechanical behavior of disordered alloys with reasonable computational cost, while maintaining statistical accuracy up to a specified correlation range. However, despite its efficiency, the method remains a finite-size approximation that may not capture long-range or subtle ordering effects. Nonetheless, SQS models are widely used in the literature as a reliable and tractable representation of random alloys in HEA research [79,80,81].

For instance, finite-temperature DFT studies of refractory systems such as NbMoTa and NbMoTaW have revealed the role of short-range order in governing stability [82], while closed-loop active learning frameworks integrating DFT, thermodynamics, and ML have led to the accelerated discovery of Invar-like HEAs with ultra-low thermal expansion [83]. Nonetheless, computational challenges persist because configurational sampling in multicomponent alloys is costly, and the inclusion of magnetism, spin–orbit coupling, and local lattice distortions significantly increases computational demand [84].

### 3.2. CALPHAD Methods of HEAs

Complementary to DFT, CALPHAD (CALculation of PHAse Diagrams) methods remain the most widely used thermodynamic approach for HEAs [85]. CALPHAD enables extrapolation of Gibbs energies from binary and ternary subsystems into higher-order composition spaces, providing phase diagrams and transformation pathways that guide alloy design [86]. The fundamental principle behind CAPLHAD is that the equilibrium state of a multicomponent material is the state that minimizes the total Gibbs free energy (G) of the system at fixed overall composition and temperature. Therefore, accurate phase prediction reduces to constructing reliable, physically grounded models of the Gibbs energy G(x,T) for each candidate phase and then performing constrained minimization subject to mass-balance (stoichiometric) constraints [87]. In practice this means (1) decomposing G(x,T) into physically motivated contributions (reference-state energies, configurational, vibrational, magnetic, electronic terms), (2) representing chemical complexity using sublattice models so that site occupancies and ordering (including non-stoichiometric behavior) are treated explicitly, and (3) fitting interaction parameters to experimental and first-principles data so the G(x,T) surfaces smoothly interpolate from binaries/ternaries into higher order systems [88].

For HEAs—which are often neither simple random solid solutions nor stoichiometric compounds—the configurational term (and its treatment via sublattices, site fractions and order parameters) becomes decisive. Small changes in sublattice occupancy or short-range ordering can alter the configurational entropy and hence shift phase boundaries, so CALPHAD models must explicitly allow multiple sublattices, off-stoichiometry end-members, and composition-dependent interaction parameters to capture HEA thermodynamics [89].

However, the lack of reliable assessed databases for multicomponent systems limits predictive accuracy. To address this, hybrid strategies combining CALPHAD with ML and Bayesian optimization have emerged, offering accelerated prediction with quantified uncertainty [90,91]. Recent applications include the thermodynamic modeling of septenary alloys such as NiAlCoCrCuMnTi, where CALPHAD coupled with DFT and ML successfully reproduced experimental phase stabilities [92].

Statistical mechanical approaches based on cluster expansion (CE) coupled with Monte Carlo (MC) simulations offer another route for linking atomistic energetics with finite-temperature phase behavior. CE reduces the configurational complexity of multicomponent systems into effective Hamiltonians trained on DFT datasets, enabling MC sampling of order–disorder transitions, SRO, and spinodal decomposition [93]. Newer CE frameworks employ transfer learning across alloy families, allowing efficient prediction of formation energies and magnetic properties with reduced DFT training requirements [94]. Furthermore, GPU-accelerated MC simulations have enabled billion-atom sampling in systems such as FeCoNiAlTi and MoNbTaW, revealing nanoscale ordering and emergent nanophases that would be inaccessible through traditional methods [95].

Molecular dynamics (MD) simulations complement these thermodynamic tools by resolving atomic-scale mechanisms of deformation, diffusion and defect evolution under realistic loading conditions. Classical embedded-atom method (EAM) and modified EAM potentials have been widely applied, but their reliability diminishes in multicomponent HEAs. The introduction of ML interatomic potentials (MLIPs), such as Gaussian approximation potentials (GAP) and spectral neighbor analysis potentials (SNAP), trained on DFT datasets, now allows MD simulations to achieve near-first-principles fidelity at system sizes of millions of atoms [96]. Machine learning interatomic potentials (MLIPs) can reproduce the results of quantum-mechanical methods, such as density functional theory (DFT) with errors comparable to, or smaller than, the intrinsic uncertainty of DFT itself. Quantitatively, this typically corresponds to energy deviations of about 1–10 meV/atom and force errors of 0.01–0.05 eV/Å, values within the range of DFT numerical convergence. Modern MLIP frameworks such as GAP, SNAP, DeePMD, NequIP, and MACE have demonstrated such accuracies across diverse materials systems, while enabling simulations of up to millions of atoms, far beyond the computational limits of direct first-principles calculations. Consequently, “near–first-principles fidelity” signifies that MLIPs retain almost the same predictive quality as DFT, but at a computational cost closer to classical force fields [97,98].

Recent work has shown how MLIPs accurately capture dislocation nucleation, defect interactions, and strengthening mechanisms in alloys such as AlCoCrFeNi, providing insight into experimentally observed mechanical responses [99].

The rapid advancement of ML in HEA research extends beyond MLIPs into property prediction, surrogate modeling, and inverse design. ML pipelines typically extract descriptors from atomic size mismatch, electronegativity differences, and valence electron concentrations to predict phase formation, hardness, and ductility [100]. Ensemble learning and deep neural networks have achieved robust performance in classifying BCC, FCC, and mixed phases and in predicting yield strengths across diverse HEA chemistries [101]. Recent studies demonstrate that feature importance analyses, using methods such as SHAP, allow interpretability of ML models, thereby correlating abstract descriptors with underlying physical mechanisms [102]. Data augmentation using generative adversarial networks (GANs) further expands limited datasets, improving model generalizability for systems with scarce experimental data [103]. Importantly, large, curated datasets containing thousands of structures with elastic properties have been released, providing benchmarks for future ML development [104].

The integration of computational tiers into multiscale frameworks represents the next frontier in HEA research. High-throughput DFT and CALPHAD calculations can be used to train surrogate CE or ML models, which in turn inform MLIPs for large-scale MD simulations. These atomistic results feed into phase-field models and continuum-scale finite-element simulations, ultimately predicting mechanical or functional performance at the macroscopic scale [105]. The coupling of such models with evolutionary algorithms creates closed-loop design systems, where promising compositions are continuously refined against both computational and experimental data [106].

A particularly critical area of progress concerns the explicit treatment of short-range order, which strongly influences stacking-fault energies, slip behavior, and diffusion kinetics. Models that ignore SRO often mispredict phase boundaries and mechanical responses. Recent CE + MC and MLIP studies show that even subtle degrees of SRO in CrCoNi-based alloys substantially alter predicted plasticity mechanisms [107]. Furthermore, billion-atom Monte Carlo simulations have identified nanophase ordering phenomena in refractory HEAs that directly correlate with observed high-temperature mechanical strength.

### 3.3. Limitations Inherent in Current Computational Frameworks of HEAs and Future Directions

The integration of CALPHAD with ML has become a leading strategy to accelerate exploration of HEAs. However, despite promising demonstrations, current CALPHAD–ML frameworks suffer from a set of systematic limitations that constrain predictive fidelity, generalizability across compositional/processing space, and utility for experimental guidance. This section summarizes the dominant failure modes—data limitations, model and representation issues, extrapolation and uncertainty, thermodynamic model inconsistency, and practical/algorithmic bottlenecks—and points to mitigation directions supported by recent literature.

The most fundamental limitation is sparse, heterogeneous, and biased data coverage in both thermodynamic databases and experimental repositories. CALPHAD parameterizations and ML training sets are often assembled from a mixture of binary/ternary assessments, first-principles calculations, and scattered experimental measurements. By construction, these sources do not uniformly sample the high-dimensional composition–temperature space relevant to multicomponent HEAs. As a result, ML models trained on available CALPHAD outputs or experiments inherit substantial sampling bias: predictions are reliable near well-studied compositions, but can be catastrophically wrong when interpolating among many lightly sampled principal elements or when crossing into regions where higher-order interactions dominate. Recent reviews emphasize that limited and uneven data remains the primary bottleneck for ML-driven HEA discovery [108].

Computational approaches to HEAs face a dual burden of scale and physics: while CALPHAD and mean-field thermodynamic extrapolations remain indispensable for exploring vast multicomponent composition spaces, their predictive power is limited by sparse low-order assessments, incomplete descriptions of non-configurational entropic contributions and uncertain extension rules for higher-order interactions—problems that cause systematic errors when short-range order, magnetic and vibrational effects become important [109].

First-principles methods (DFT, cluster expansions, ab initio MD) provide crucial atomistic insight into site preferences, electronic/magnetic contributions and order–disorder transitions, but they struggle with the combinatorial explosion of configurations, the expensive evaluation of vibrational and magnetic free energies, and with sampling realistic local-lattice distortions at experimentally relevant temperatures [110].

In addition, grain boundary (GB) control is a persistent, universal bottleneck when translating first-principles predictions into reliable, real-world alloys. GBs act as the decisive loci for chemical segregation, decohesion, embrittlement, corrosion initiation and damage accumulation, so that bulk-focused design rules alone often fail to predict service behavior [111]. In HEAs, this challenge is strongly amplified: the multicomponent chemistry produces non-trivial GB compositions, local short-range order and emergent “high-entropy” interfacial thermodynamics that can either stabilize or destabilize boundaries in ways not seen in dilute alloys. In this way, GBs may become sinks for deleterious species or, conversely, sites of unexpected strengthening depending on temperature, processing and local chemistry [112]. Practically, this means (a) alloy screening must explicitly include GB segregation and cohesion metrics, (b) thermomechanical processing (and additive manufacturing) must be tuned to control GB character distribution and solute partitioning, and (c) experimental validation at the nanoscale (APT, TEM) is essential because predicted bulk indicators can miss interface-driven failure modes [113].

Capturing chemical short-range order, correlated local strains and heterogeneous defect energetics—phenomena recently shown to control phase stability and mechanical response—requires methods that go beyond averaged Hamiltonians (e.g., many-body potentials trained on DFT, advanced Monte Carlo schemes and embedded cluster approaches) and benchmarks against in situ experiments [114].

Machine learning and high-throughput frameworks are accelerating discovery by constructing rapid surrogate models for formation energies, phase classification and site-resolved properties, yet these data-driven models inherit biases from limited training sets, lack robust uncertainty quantification for extrapolation across chemistries and often fail to respect fundamental physical constraints unless hybrid physics-informed architectures are used [115].

Promising mitigation strategies already emerging in recent literature include curated, open HEA databases with explicit parameter covariance and provenance; transfer- and few-shot learning to leverage binary/ternary data while controlling extrapolation error; integrated Bayesian UQ that propagates CALPHAD parameter uncertainty into ML predictions; physics-informed representations that encode local environment and many-body interactions; active-learning experimental loops that prioritize regions of high model uncertainty; and tighter integration of mobility/kinetics databases to predict non-equilibrium outcomes [116,117]. Adoption of these practices, together with community standards for database versioning and benchmark tasks, will be required to move CALPHAD–ML frameworks from promising tools to robust engines for HEA design. The most promising future directions therefore emphasize integrated multiscale workflows—coupling CALPHAD, physics-aware ML, DFT/cluster-expansion, atomistic dynamics and kinetic solvers—accompanied by systematic efforts to expand high-quality thermochemical databases, standardized active-learning data generation, and rigorous uncertainty propagation, so that predictive alloy design moves from retrospective interpretation to prescriptive, process-aware optimization [118]. Table 1 summarizes the strengths, limitations and typical outputs of the computational methods applied to HEAs.

## 4. Processing Techniques

Processing of HEAs is no longer an exploratory afterthought, but the principal axis that determines which of the many promising compositions become useful materials. Processing routes—liquid melting and casting, powder metallurgy and mechanical alloying, additive manufacturing, thermomechanical treatments including severe plastic deformation (SPD), surface techniques (sputtering, cladding, thermal spray), and targeted heat-treatment schedules—do far more than densify material: they determine phase constitution (single-phase vs. multiphase; FCC/BCC/B2/L1_2_/σ), chemical homogeneity and short-range order, defect content (dislocation density, vacancies, porosity, microcracks), grain and cell-wall architectures, and microscale chemical segregation patterns that govern corrosion, fatigue and creep performance. The latest literature emphasizes that success in HEA engineering depends on integrating (i) feedstock design and preparation of each method, (ii) process physics (solidification, diffusion, deformation), and (iii) data-guided thermodynamics/kinetics modeling to define reproducible process windows that will produce targeted microstructural hierarchies [119,120,121,122]. Table 2 compares the processing routes for HEAs, by highlighting their microstructure, benefits, and challenges.

### 4.1. Melt-Based Routes and Rapid Solidification

Traditional melt processing—arc melting, induction melting and vacuum melting—remains the gateway to bulk HEA production, since it scales readily and accommodates dense, large billets. However, the intrinsically wide solidification ranges and complex multi-element partitioning tendencies of many HEAs, cause micro-segregation, inter-dendritic intermetallic formation and, in extreme cases, hot-cracking. Rapid solidification approaches (melt spinning, splat quenching, laser remelting) compress the timescale for partitioning, enabling supersaturated solid solutions, high defect concentrations and chemically disordered states that can be later evolved into desirable precipitate-strengthened microstructures by annealing. Directional solidification and controlled cooling have been used deliberately to create eutectic HEAs, in which lamellar B2/L1_2_ or FCC/BCC architectures provide intrinsic microstructural heterogeneity that is beneficial for strength–toughness balance. Recent studies show that combining directional solidification with modest thermomechanical processing can optimize the lamellar spacing and phase connectivity that underpin high fracture resistance.

Eutectic HEAs are a special subset of HEAs that solidify via a eutectic reaction, typically forming two distinct solid phases (often with different crystal structures) in a lamellar or regular composite microstructure. This dual-phase structure is desirable because it combines strength and ductility-one phase provides hardness/strength, the other toughness/ductility. The lamellar B2/L1_2_ structure provides a balance of strength and ductility, and is one of the most widely reported and studied eutectic HEA microstructures. The B2 phase is typically hard and brittle, while the L1_2_ phase is ductile and tough. The B2 is ordered body-centered cubic (BCC) structure (like NiAl-type), while the L1_2_ is ordered face-centered cubic (FCC) structure (like Ni_3_Al-type). A characteristic example system is AllCoCrFeNi_2.1_ [123]. Another common pairing is FCC/BCC or FCC/B2, depending on whether the BCC phase is ordered (B2) or disordered. The FCC phase contributes to ductility and the BCC or B2 phase contributes to strength. Lamellar morphology results from the eutectic reaction during solidification.

While B2/L1_2_ and FCC/BCC (or FCC/B2) are the most typical lamellar architectures in eutectic HEAs, other pairings have been reported, though less frequently. For example:

- BCC + intermetallic (e.g., σ-phase or μ-phase): can occur in Cr- or Mo-rich HEAs, providing hardness, but less ductility. Characteristic example is the CrFeCoNi-based systems [124].
- FCC + Laves phase: it is mostly observed in systems with refractory or transition metals (e.g., V, Nb, Mo), e.g., AlCoCrFeNiNbₓ [125].

To summarize, the dominant concerns in melt processing are (a) controlling cooling rate and thermal gradients to avoid deleterious phase fractions, (b) managing macrosegregation in large castings, and (c) designing post-cast homogenization schedules that dissolve undesirable interdendritic phases without triggering excessive grain growth [126,127,128].

### 4.2. Powder Metallurgy, Mechanical Alloying and Sintering Strategies

Solid-state powder routes have evolved as the most flexible means to produce compositionally complex HEAs with minimal macrosegregation. High-energy mechanical alloying (MA) is particularly powerful because it creates nanocrystalline or amorphous precursor powders with intimate elemental mixing and high defect densities, enabling the synthesis of alloys that are difficult to obtain by melting (e.g., compositions containing highly reactive or refractory elements). Consolidation techniques—spark plasma sintering (SPS), hot pressing, hot isostatic pressing (HIP) and field-assisted sintering—are now routine and can achieve near-full densification at lower overall thermal exposure than conventional sintering, preserving refined grains and enabling controlled precipitation. Hybrid flows that combine MA → SPS → thermomechanical processing or MA → powder spheroidization → additive manufacturing are increasingly common, because they merge the mixing fidelity of MA with the geometric freedom of AM. Recent systematic comparisons highlight how milling energy, ball-to-powder ratio, SPS current/pressure schedules and dwell temperatures map onto final grain size, residual porosity, and precipitation states, allowing process maps that predict mechanical property envelopes for different HEA chemistries. Powders also enable dispersion strengthening (ceramic dispersoids, in situ nitrides/carbides) and graded compositions for functionally tailored parts [129,130,131,132].

### 4.3. Additive Manufacturing of HEAs: Opportunities and Limitations

Additive manufacturing (AM) has arguably been the single most disruptive processing advance for HEAs in the last decade because its layerwise thermal histories and extremely high local cooling rates frequently produce microstructures that are inaccessible by conventional casting or PM. Powder-bed fusion (PBF-LB/PBF-EB), directed energy deposition (DED), binder-jetting plus sintering, and solid-state approaches such as additive friction stir deposition (AFSD) each impose distinct solidification and reheating patterns that control cell/dendrite morphology, segregation, and residual stresses. AM can create strong directional anisotropy, cellular substructures with solute-enriched cell walls, and high dislocation densities that, when tempered by post-build heat treatments, yield to unusual strength–ductility combinations.

The dominant cause of the pronounced directional anisotropy observed in AM-processed HEAs is epitaxial grain growth during successive layer-by-layer melting and solidification. Each new melt pool solidifies onto the partially melted/solid substrate beneath because nucleation density is low under rapid solidification. Also, due to the strong directional heat extraction (large temperature gradient G) along the build direction, existing grains grow across layer boundaries and evolve into elongated/columnar structures with a preferred crystallographic orientation aligned with the build direction [133].

HEAs can further accentuate these effects because their complex, multi-element chemistry modifies solidification behavior. Slowed diffusion and complex partitioning can favor persistence of dendritic/columnar morphologies and reduce the tendency for post-solidification recrystallization that would randomize texture. In practice, AM-HEA studies frequently report epitaxial columnar grains and build-direction texture, unless specific processing strategies (such as remelting or thermal control) [134] are used to promote new nucleation or equiaxed growth [135].

Challenges are substantial: hot-cracking susceptibility increases with wider mushy zones and non-ideal alloy thermodynamics; porosity and keyholing are sensitive to energy density and powder characteristics; and the interaction of residual stress, surface roughness and micro-segregation complicates fatigue and corrosion behavior. Accordingly, recent literature emphasizes three practical themes: choosing AM-friendly chemistries (narrow solidification ranges), optimizing scan strategies and pre/post-heat treatments to remove harmful ordering/segregation, and combining AM with targeted post-AM thermomechanical processing to homogenize and tailor precipitate populations. For refractory HEAs, AM has shown promise but faces additional hurdles from vaporization, large melting temperatures, and thermal mismatch with common substrates [3,136,137,138].

### 4.4. Thermomechanical Processing, Severe Plastic Deformation (SPD) and Texture Control

Thermomechanical processing (rolling, forging, extrusion) remains central to producing structural HEAs with balanced strength and ductility, because it can refine grain size, alter texture and stimulate beneficial precipitation/order phenomena. SPD techniques—high-pressure torsion (HPT), equal-channel angular pressing (ECAP), accumulative roll bonding (ARB) and cryo-rolling—further extend the microstructural lever arm by producing ultrafine-grained or nanostructured states and extremely high dislocation densities. These states often show accelerated diffusion-controlled transformations and can stabilize metastable phases that contribute to strain-hardening. SPD is also a viable pathway for refractory HEAs where conventional thermomechanical routes produce brittle behavior. By producing high defect densities and ultrafine grains, SPD can improve ductility and provide templates for controlled precipitation during subsequent annealing. The experimental literature now provides process–property maps linking SPD strain, annealing temperature, and final phase fractions for a range of HEA chemistries, giving a practical nucleus for designing combined SPD–anneal sequences. However, SPD processes can create strong textures whose anisotropy must be considered when designing components for fatigue or directional loading [139,140,141,142].

### 4.5. Surface Engineering, Thin Films and Coatings

Gas-phase and line-of-sight deposition methods (magnetron sputtering, pulsed laser deposition, molecular beam epitaxy) afford extreme compositional control and very high effective cooling rates, enabling single-phase nanoscale solid solutions and novel oxide or nitride high-entropy derivatives. Thermal spray and laser cladding translate HEA chemistries into wear- and corrosion-resistant overlays on conventional engineering substrates; graded or functionally layered feedstock designs mitigate thermal mismatch and residual stress. The distinctive advantage of surface approaches is the decoupling of bulk and surface performance: a hard, chemically robust HEA coating can be applied to a ductile substrate, delivering surface functionality with manageable cost. Recent surveys emphasize feedstock preparation (spheroidized powders, pre-alloyed feedstock), process control (particle temperature/velocity in thermal spray), and post-deposition treatments to reduce porosity and residual tensile stresses, that otherwise compromise adhesion and fatigue life [143].

### 4.6. Heat Treatment, Homogenization, Ordering and Precipitate Engineering

Across processing routes the final microstructure almost always depends on tailored heat treatments. Homogenization reduces inter-dendritic segregation inherited from casting or layerwise AM, but it competes with grain growth; short, high-temperature solution treatments followed by controlled aging/precipitation steps (L1_2_ in FCC matrices; B2 or ordered phases in BCC matrices) are widely used to create coherent nanoscale strengthening phases. For alloys designed for oxidation or corrosion resistance, modest anneals often improve passive film formation by evening out surface chemistry and relieving near-surface strain. The kinetics of ordering and precipitation in multicomponent systems can differ markedly from binary alloys—phase-field and CALPHAD-based modeling, now commonly coupled with experiments, provide essential guidance on treatment temperatures and times to achieve metastable but desirable distributions of ordered domains or nanoscale precipitates [144,145,146,147].

### 4.7. Process-Aware Alloy Design: Modeling, Machine Learning and High-Throughput Workflows

The explosion of possible compositions in HEA space makes empirical screening impractical. Accordingly, modern processing strategies nearly always include computational elements. CALPHAD and phase-field models provide physical priors about phase stability, solidification ranges and diffusion pathways, while machine-learning surrogates trained on experimental and simulated databases accelerate screening for AM-compatible chemistries, SPS schedules that avoid grain growth, or SPD + anneal sequences that target dual-phase microstructures. High-throughput experiments—combinatorial thin films, rapid powder synthesis, automated AM parameter sweeps—are increasingly combined with ML to identify process windows that minimize hot cracking, porosity and deleterious intermetallic formation. This convergence of computation and experiment is already producing predictive frameworks for process design, rather than reactive trial-and-error optimization [148].

### 4.8. Challenges, Open Problems and Outlook

Despite the rapid maturation of processing science for HEAs, several persistent challenges remain. First, the translation of laboratory-scale processing recipes to industrial scales encounters segregation and thermal-mass problems that are not captured in small samples. Second, the long-term mechanical performance of many process-engineered microstructures (fatigue under complex multiaxial loading, creep at elevated temperature, stress-corrosion cracking in service environments) is still insufficiently charted. Third, refractory HEAs offer tantalizing high-temperature performance, but require new strategies for feedstock production, vapor-pressure management in AM, and oxidation protection. Finally, the community needs standardized process reporting and shared databases, so that models trained on one laboratory’s data generalize to others.

Thus, a major challenge in HEA research is the lack of standardized process reporting, which hampers reproducibility and meaningful comparison between studies. Critical parameters that are often inconsistently reported include the exact elemental composition and purity of the starting materials, the melting and casting conditions (such as method, atmosphere, temperature, and number of remelts), and mechanical processing details like rolling or forging temperatures, strain rates, and inter-pass treatments. Heat treatment protocols—including solution treatment temperatures, durations, cooling methods, and aging conditions—are also frequently underreported. In powder-based processing, factors such as powder production method, particle size distribution, compaction pressure, and sintering atmosphere strongly influence the final microstructure. Additionally, environmental conditions during processing and characterization, including oxygen and moisture levels and testing protocols, play a critical role in material properties. Without consistent reporting of these parameters, it is difficult to reproduce experiments, compare results across different studies, or establish reliable structure–property relationships for HEAs.

Processing of HEAs also remains bottlenecked by tightly coupled materials-physics and manufacturing constraints. Additive manufacturing (AM) unlocks unprecedented compositional and geometric freedom, but simultaneously amplifies problems of compositional segregation, keyhole/porosity formation, nonequilibrium phase selection and anisotropic microstructures-issues that are accentuated for refractory and high-melting HEAs, where thermal management and feedstock chemistry critically determine phase stability [149].

Powder-based routes and mechanical alloying provide scalable feedstocks, but face powder-quality, oxygen pickup and contamination trade-offs that alter kinetics and embrittle otherwise ductile chemistries, while wire- and bulk-based deposition methods (WAAM, DED) struggle to deliver consistent microstructural homogeneity at production scales [150].

Severe plastic deformation and thermomechanical processing can produce ultrafine, gradient and heterostructured HEAs with superior strength–ductility envelopes, yet controlling grain-boundary chemistry, retained strain energy and post-deformation thermal stability require tightly coupled process models and in-process monitoring, which is still immature [151].

Rapid solidification and AM-induced metastability open pathways to desirable microstructures but also generate metastable phases and residual stress states that complicate downstream heat treatments and property reproducibility, calling for integrated process–structure–property frameworks and standardized post-processing protocols [152].

The path forward is one in which compositional design and processing design are co-developed: process-aware alloy discovery that prescribes not just composition but a validated manufacturing route, and process-aware standards that allow HEAs to move from laboratories into certified components [129,141,144]. Future directions should therefore prioritize: (i) feedstock engineering (controlled powder/wire chemistries, low-oxygen routes), (ii) physics-aware process models and closed-loop in situ sensing for defect mitigation and thermal control, (iii) scalable post-processing (thermomechanical and SPD combinations) that stabilize targeted microstructures, and (iv) cross-platform benchmark studies to quantify reproducibility and lifecycle costs, so that HEA processing moves from laboratory demonstrations to industrially robust manufacture [153].

## 5. Mechanical Properties

The mechanical behavior of HEAs has become one of the central interrogations driving both fundamental studies and application-driven research, because the unusual compositional complexity that defines HEAs simultaneously generates novel deformation mechanisms and a wide dispersion of achievable property combinations. From the seminal conceptual papers that introduced multi–principal-element alloys and documented single-phase solid solutions [141,142], a profusion of work has shown that HEAs can exhibit combinations of high strength, exceptional ductility, temperature-insensitive flow, and useful fracture/toughness performance that are rarely observed in conventional binary or ternary alloys [102,154,155,156]. This section synthesizes the current understanding of HEA mechanical properties, organized by regime and closes with examples tying properties explicitly to processing and design strategies.

Recent developments in HEA design have focused on improving the mechanical properties through the incorporation of interstitial elements like carbon, nitrogen, and boron, which have been found to enhance both the strength and high-temperature stability of HEAs.

The basic mechanisms covering the mechanical properties of HEAs are the following:

- Solid-solution strengthening in HEAs arises because the multiple principal elements create local chemical and atomic size fluctuations, which hinder dislocation motion.
- Grain-refinement (via severe plastic deformation or equal channel angular pressing (ECAP)) further enhances strength through the Hall–Petch effect.
- Precipitation or coherent second-phases (e.g., carbides, nitrides, borides) can provide additional hardening, though this is less common in classical “single-phase” HEAs.
- Interstitial alloying (C, N, O, B, H) is emerging as a powerful route to raise strength, by introducing local lattice distortions, interstitial-atom/dislocation interactions, and in some cases micro-alloyed precipitates [157].
- Literature suggests that interstitial alloying can in some cases partially circumvent the classic strength–ductility trade off, i.e., increasing strength without a precipitous drop in ductility [158,159].

### 5.1. Yield, Tensile Strength and Ductility: Typical Envelopes and Exemplar Alloys

The yield strength and tensile strength of HEAs are among their most notable features. These properties arise from the atomic-level interactions in the solid-solution phase, which disrupt dislocation motion and contribute to the high strength of these materials. Traditional HEAs such as CoCrFeNiMn and FeNiCoCr exhibit yield strengths ranging from 700–1000 MPa, while tensile strengths are typically in the range of 1000–1500 MPa. However, the incorporation of interstitial elements has led to significant improvements. Recent developments regarding interstitial strengthening claim that the addition of carbon (C) and nitrogen (N) has been found to enhance the solid-solution strengthening mechanism, leading to higher yield and tensile strengths [160]. For instance, a study by Zhang et al. on a FeNiCoCr–C HEA showed a yield strength increase to 1300 MPa and a tensile strength increase to 1700 MPa, compared to 1100 MPa and 1400 MPa, respectively, in the base FeNiCoCr alloy [161]. A meta-analysis of the yield strength across various HEA systems indicates that alloys with higher contents of refractory metals (e.g., Mo, W) tend to show superior strength, particularly at high temperatures, due to the presence of stronger atomic bonds.

While HEAs are known for their strength, their ductility and elongation to failure can vary significantly, depending on composition and microstructure. Generally, HEAs with high entropy elements exhibit good ductility, but the addition of interstitials or the presence of certain phase structures can reduce elongation, a phenomenon observed in several studies on interstitial-strengthened HEAs. Recent observations on ductility vs. strength consistently highlight a trade-off between the strength and ductility of HEAs. For example, in the study by Zhang et al. [161], the addition of carbon to a FeNiCoCr alloy significantly improved yield strength, but led to a reduction in elongation to failure, with values dropping from 50% to 35%. At ambient temperatures many single-phase FCC HEAs such as CoCrFeMnNi and its near-derivatives, combine moderate-to-high ductility with modest yield strength (~200–500 MPa in conventionally processed material (cast + homogenized + annealed (i.e., a fully recrystallized, coarse-grained FCC structure), without further strengthening treatments such as cold working, precipitation, or nanostructuring)) and significant strain-hardening capacity, while BCC and multi-phase HEAs often trade ductility for much higher strength (often >800–1000 MPa) [119,120,154].

The more recent surge of alloy classes—eutectic HEAs (EHEAs), precipitation-strengthened HEAs (e.g., L1_2_-type precipitates in FCC matrices), and refractory HEAs (RHEAs)—has broadened the envelope: eutectic and precipitation-strengthened alloys can simultaneously achieve ultra-high yield strengths (often >1 GPa) with non-negligible ductility when their microstructural length scales and phase connectivity are optimized [130,155,162,163]. Refractory HEAs, engineered for high-temperature structural use, generally yield exceptional high-temperature strengths, but can be brittle at room temperature unless nanoscale design (grain refinement, engineered precipitates, or controlled disorder) and specific processing (e.g., SPD or tailored heat treatments) are applied to restore ductility [156,164]. Several high-impact experimental demonstrations illustrate these trends: the phase-selectively recrystallized eutectic HEA achieves near-gigapascal true stresses with tens of percent uniform elongation by designing soft-phase continuity and hard-phase skeletons; refractory compositions such as AlNbTaTiVZr derivatives show tunable A2/B2 microstructures with markedly improved room-temperature ductility after careful thermomechanical processing [156,163].

### 5.2. Deformation Mechanisms: Dislocations, Twinning, TRIP and Phase Transformations

HEAs display a rich palette of operative deformation mechanisms that depend strongly on crystal structure, stacking fault energy (SFE), solute effects, and microstructural constraints. In FCC HEAs with relatively high SFE the classical dislocation slip on multiple systems dominates and produces sustained strain hardening via dislocation accumulation and complex forest interactions. Alloys with moderate-to-low SFE activate deformation twinning (TWIP-like behavior) which contributes to high work hardening and delayed necking. Alloys deliberately designed with a metastable phase field can undergo stress-induced martensitic transformations (TRIP-like behavior) that markedly increase both work hardening and ductility [165,166,167]. In BCC and refractory HEAs, non-planar screw dislocation core effects, temperature-dependent kinetic processes, and thermally activated cross-core motion control flow. These mechanisms can give rise to high yield strengths, but also to strong strain-rate and temperature sensitivities in flow stress [156,168].

Non-planar dislocation cores in HEAs—arising from severe lattice distortion and chemical disorder—play a crucial role in governing their macroscopic mechanical response. These complex cores increase the lattice friction, thereby reducing dislocation mobility and promoting thermally activated glide. As a result, HEAs exhibit high strength, pronounced temperature, strain-rate sensitivity, and enhanced work-hardening behavior, like that observed in BCC metals. Atomistic simulations and experimental studies have shown that such non-planar cores can spread over multiple {111} planes in FCC HEAs like CrMnFeCoNi, leading to heterogeneous dislocation motion and cross-slip at elevated temperatures [169,170,171]. Consequently, the intrinsic lattice friction associated with non-planar core structures provides a fundamental mechanism linking atomic-scale disorder to the unique thermomechanical properties of HEAs.

Recent microstructural and in situ deformation studies provide direct evidence for coupled mechanisms-e.g., dislocation channels nucleating at solute-rich cell walls in additively manufactured microstructures, twin-dislocation interactions that produce exceptional strain hardening in CoCrNi-based alloys, and phase transformation fronts in eutectic/heavily alloyed systems that transfer load across phase boundaries-emphasizing that multiscale heterogeneity is often the enabler of desirable mechanical combinations rather than an obstacle [130,172].

### 5.3. Strain Hardening, Work-Softening and Toughening Strategies

Sustained strain hardening is a prerequisite for high uniform elongation and toughness. HEA design strategies to increase strain hardening include decreasing SFE to promote twinning, engineering metastability to enable TRIP, embedding nanoscale coherent precipitates (L1_2_, B2) to raise the initial yield while preserving subsequent work hardening, and designing bimodal or hierarchical grain-size distributions to create strain-partitioning and back-stress hardening. Notable experimental reports document that finely distributed L1_2_ precipitates (created by controlled aging of FCC HEAs) generate yield strengths exceeding 1 GPa, while retaining appreciable ductility due to continued dislocation multiplication and precipitate-matrix interactions [162]. Similarly, eutectic HEAs with optimized lamellar spacings can reconcile strength and toughness by promoting crack deflection, crack-tip blunting and ductile phase pull-out, as shown in multiple recent studies [130,155]. Toughness measurements and fracture mechanics tests are now appearing more often in the literature, revealing that microstructural connectivity (continuous ductile ligaments vs. brittle intermetallic networks) is a decisive factor in determining K_IC_ and crack growth resistance, and that process pathways which avoid brittle, continuous inter-dendritic phases (via homogenization or tailored solidification/AM strategies) markedly improve fracture performance [155].

### 5.4. Fatigue and Cyclic Deformation: Initiation, Small-Crack Growth and Life Prediction

Fatigue research on HEAs, while expanding, remains less comprehensive than monotonic tensile testing. Fatigue crack initiation is often associated with surface defects, inclusions, or localized microstructural heterogeneities such as solute-rich cell walls in AM parts; microstructural features that promote uniform plasticity (e.g., stable twinning, fine coherent precipitates) tend to delay crack initiation and slow small-crack growth [173]. For CoCrFeMnNi and CoCrNi derivatives, systematic fatigue studies indicate that cold-worked plus annealed microstructures and shot-peened surfaces extend fatigue life by increasing the near-surface compressive residual stress and by homogenizing the near-surface chemistry [174]. Recent reviews and experimental contributions document case studies where process modifications (AM parameter optimization, surface treatments, or composite/dispersion strategies) significantly improved fatigue endurance limits, but they also emphasize the need for standardized testing and long-term data to enable reliable life-prediction models in engineering contexts [173,175].

### 5.5. High-Temperature Performance and Creep Resistance

One less-studied but increasingly important domain for HEAs is their mechanical performance at elevated temperature, i.e., yield strength retention at 500–1000 °C, creep behavior and microstructural stability under thermal exposure. Refractory HEAs show remarkably high yield strengths at temperatures where conventional alloys soften, and some compositions present oxidation-stable surface scales with good creep resistance. Nevertheless, high diffusivity of particular species, short-circuit paths along complex grain-boundary chemistries and the emergence of deleterious phases under long exposures remain critical challenges [156,163].

The addition of interstitial elements (C, N) has been shown to enhance creep resistance at elevated temperatures. Creep studies on several recent HEA variants show competitive minimum-creep-rate regimes compared with conventional superalloys at certain stress–temperature windows, but overall, the high-temperature alloy design problem for HEAs demands careful management of phase stability, oxidation kinetics and microstructural coarsening [172,176].

For example, Sun et al. demonstrated that FeNiCoCr–N HEAs retained excellent elongation (30%) even at temperatures exceeding 900 °C, suggesting that nitrogen strengthened grain boundaries without sacrificing ductility at high temperatures. Many refractory-HEAs (including elements such as W, Mo, Nb, V) show better high temperature strength/creep resistance than classic FeNiCoCr based HEAs, thanks to stronger atomic bonds and improved stability of the solid-solution phase at elevated temperature. Interstitial-strengthened HEAs may offer further advantages at high temperature. Reduced diffusion (sluggish diffusion) and stronger lattice may suppress creep mechanisms. The study on equiatomic CoCrFeNiMn provides some quantitative data [177]. Precipitation-strengthened HEAs (e.g., L1_2_-containing Ni-rich derivatives) use classical high-temperature strengthening concepts but benefit from sluggish diffusion or complex partitioning effects that can extend the useful temperature range of coherent precipitates [178,179].

### 5.6. Fracture Mechanics: Crack-Tip Processes and Microstructural Design for Toughness

Fracture behavior in HEAs is strongly microstructure dependent. Alloys that are nominally single-phase but contain severe chemical short-range order, coarse precipitates, or brittle intermetallics often exhibit low toughness. On the contrary, microstructures engineered with ductile matrices and dispersed hard phases (lamellar EHEAs, bimodal grain structures, or coherent precipitate arrays) show improved resistance to crack initiation and propagation [130,155,180,181]. Fractographic and in situ microscopy studies reveal common toughening mechanisms: crack blunting and bridging by ductile ligaments, decohesion-limited fracture in brittle networks, and load transfer across phase boundaries in dual-phase microstructures. These observations motivate processing strategies (directional solidification, controlled aging, phase-selective recrystallization, SPD) that intentionally produce microstructural topologies favorable to energy dissipation at growing cracks [130,164,182].

### 5.7. Size Effects, Nanostructuring and Interface Engineering

Grain-refinement and nanostructuring through SPD, severe cold rolling, or MA + SPS routes produce ultrafine-grained HEAs with dramatically raised yield strength through the Hall–Petch effect, sometimes at the expense of ductility. Careful grain-boundary engineering (bimodal or hierarchical grain-size distributions) can circumvent this tradeoff by enabling distributed plasticity and back-stress hardening [164,183,184]. Interface engineering—through coherent precipitates, designed phase boundaries in eutectic microstructures, or graded coatings—provides another avenue to tailor dislocation transmission, crack blunting and thermal stability. Experimental results in both bulk and thin-film HEAs demonstrate that interfacial character and length scale often dominate mechanical response when dimensions approach sub-micron regimes [183,184].

### 5.8. Modeling, Data Integration and Predictive Property Design

In first-principles studies of multicomponent and high-entropy alloys, the dominant approaches for predicting elastic and related mechanical properties are: (i) explicit supercell approaches that model chemical disorder via Special Quasi-Random Structures (SQS) (see more details in Section 4.1) and large supercells, (ii) effective-medium methods such as the Korringa–Kohn–Rostoker or other Coherent-Potential-Approximation (KKR-CPA/CPA) families that treat substitutional disorder in an averaged way, and (iii) high-throughput/machine-learning accelerations built on DFT databases and interatomic potentials to extend composition space [185,186].

Among those methods, the mechanical response of multicomponent high-entropy alloys is most reliably obtained from first-principles density-functional-theory (DFT) by applying infinitesimal (and finite) lattice deformations and extracting elastic coefficients either from the quadratic dependence of the total energy on strain or directly from the stress tensor. In the energy-strain route [187] the central relation is commonly written as ΔE = V_01/2_∑i_,j_C_ij_ε_i_ε_j_, from which the independent single-crystal C_ij_ are obtained by fitting ΔE(ε) for symmetry-distinct deformation modes [188].

Recent methodological extensions include higher-order elastic constant calculations (third- and fourth order) via finite strain expansions to capture anharmonicity and large-strain responses, and stress-based differentiation schemes that compute Cauchy stresses directly to improve numerical stability. These advances permit prediction of pressure- and temperature-dependent elastic behavior and provide inputs for continuum scale models and hardness proxies [189,190].

In the energy–strain approach, small homogeneous deformations are imposed on the equilibrium lattice, and the total energy response is calculated using DFT. The elastic constants are then obtained from a quadratic fit to the strain–energy relation [191].

Computational thermodynamics (CALPHAD), phase-field modeling, dislocation-based crystal plasticity, and machine-learning surrogates are now routinely coupled to experimental campaigns to guide mechanical design. CALPHAD gives critical priors about phase stability and partitioning; phase-field models capture microstructural evolution under processing or service conditions; crystal plasticity bridges microstructure to macroscopic stress–strain behavior; and ML approaches accelerate search within the vast compositional space, identifying chemistries and process windows that yield desired combinations of strength, ductility and thermal stability [119,167]. Ongoing work is pushing toward integrated computational–experimental loops, where small-scale high-throughput testing supplies labeled data for ML models that propose candidate alloys and process schedules, which are then validated in targeted larger-scale tests. Reliable predictions still require careful treatment of magnetic, configurational and vibrational contributions when comparing them to measured mechanical properties.

### 5.9. Outlook and Research Needs

The mechanically relevant knowledge base for HEAs has expanded rapidly, but several fronts require attention: standardized fatigue and fracture datasets across processing routes and environmental conditions; long-duration creep and oxidation studies for elevated-temperature applications; systematic assessments of hydrogen-environment interactions; and industrially relevant scale-up studies that translate laboratory process controls into consistent component performance. The strongest near-term impact is likely where process-aware alloy design pairs with proven manufacturing pipelines (PM + SPS, AM with validated post-processing, SPD + anneal sequences) to deliver certified, demonstrably reliable HEA components for targeted, high-value applications [119,120,164,173].

As the latest literature highlights, the interstitial strengthening (C, N, B, O) in HEAs is a key route to elevating yield/tensile strength to 1200–1800 MPa levels, while maintaining modest ductility (~25–35%). There remains a trade-off between strength and ductility, though the magnitude of the trade-off appears less severe than in conventional alloys, thanks to the multiple strengthening mechanisms and high work-hardening capacity of HEAs. The ability to tailor stacking-fault energy (SFE) is also critical. Low SFE promotes twinning/MT, which aids ductility and work hardening, while high SFE (often induced by interstitials) may shift deformation to dislocation glide, increasing strength but lowering ductility. High-temperature performance and creep resistance remain less comprehensively quantified, but initial data are promising, especially where diffusion is suppressed and microstructures remain stable. Future research needs should prioritize systematic benchmarking of interstitial-strengthened HEAs for high-temperature creep/fatigue, quantified SFE values, and more complete databases of yield/tensile/ductility across wide compositions and processing states.

## 6. Chemical and Functional Properties

**Figure 5:**
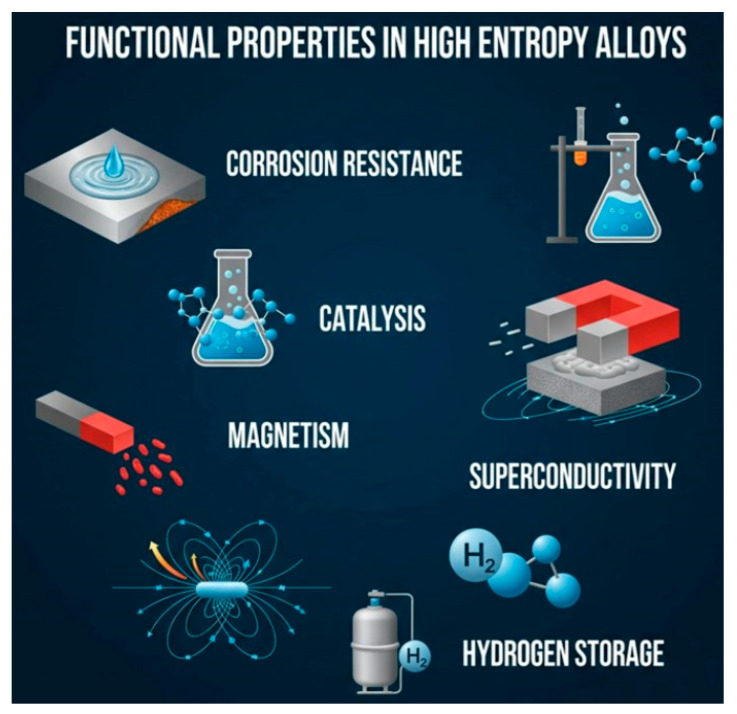
Overview of functional properties in HEAs.

Unlike conventional alloys optimized around one principal solvent, HEAs display a spectrum of emergent physical phenomena—electronic, magnetic, thermal, chemical and radiation responses-that are determined by a complex interplay of electronic structure, local lattice distortion, chemical short-range order (CSRO), and the presence of multiple local bonding motifs. The following subsections review these functional properties, summarizing principal mechanisms, giving numerical examples from representative studies, and pointing to emerging trends and outstanding challenges. Figure 5 presents an overview of the functional properties in HEAs, namely corrosion resistance, catalysis, magnetism, superconductivity and hydrogen storage and Table 3 summarizes some indicative HEA systems, and their mechanical, chemical and functional properties.

### 6.1. Electronic Transport and Thermoelectric Behavior

Electronic transport in HEAs departs from simple metallic behavior because chemical disorder dramatically increases carrier scattering and residual resistivity while sometimes suppressing the temperature dependence of resistivity. Several studies report large room-temperature resistivities and low residual-resistivity ratios (RRR), consistent with the Mott–Ioffe–Regel limit in heavily disordered metals [199,200].

HEAs typically have very high resistivities for metallic alloys, mainly because of extreme atomic disorder leading to strong electron scattering. A typical range is ρ_300K_~100–300 μΩ⋅cm. A common example is the Cantor alloy (CoCrFeMnNi) with ρ~150 μΩ⋅cm at 300 K.

The low RRR values arise due to the fact that disorder dominates even at low temperature. A typical range of RRR~1–3. HEAs are close to the Mott–Ioffe–Regel limit, where the electron mean free path becomes comparable to the interatomic spacing [201,202].

Experimental work on Ni-rich and NiCo-based multicomponent alloys shows room-temperature thermal conductivities spanning from typical metallic values (~25–100 W·m^−1^·K^−1^ in ordered transition metals) down to anomalously low values (single-digit W·m^−1^·K^−1^) in refractory, highly disordered compositions; for example, the refractory HfNbTaTiZr system exhibits lattice thermal conductivities on the order of ~9 W·m^−1^·K^−1^ at room temperature in some reports [203], and tailored compositions and microstructures have produced ultralow total thermal conductivities as low as ~0.4–0.6 W·m^−1^·K^−1^ for engineered porous or nanostructured HEA derivatives intended for thermal-insulation or thermoelectric applications [204]. From a thermoelectric standpoint, the combination of high electrical resistivity and low thermal conductivity is attractive but challenged by modest Seebeck coefficients; recent computational and experimental work aims to exploit CSRO and band-structure engineering to increase power factors while retaining phonon scattering [205,206]. Simulations and atomistic modeling have shown that increased CSRO can either raise or lower phonon heat transport depending on clustering motifs, so composition tuning remains central to optimizing thermoelectric figure-of-merit (ZT) in HEAs [203,207].

### 6.2. Thermal Conduction and Phonon Scattering

Phonon transport in HEAs is strongly affected by mass and force-constant disorder, lattice distortion and the coexistence of multiple phonon scattering channels. The “sluggish diffusion” and lattice-distortion concepts translate into strong phonon scattering and suppressed lattice thermal conductivity compared with conventional alloys of similar composition spread. Molecular dynamics and Green-Kubo calculations for refractory HEAs (e.g., Hf–Nb–Ta–Ti–Zr and Mo–W–Ta–Ti–Zr variants) find that chemical short-range order can increase or decrease thermal conductivity by modifying high-frequency phonon states; one atomistic study quantified κ ≈ 9 W·m^−1^·K^−1^ for an equilibrated HfNbTaTiZr sample and showed marked sensitivity of κ to SRO [20,208]. Experimental reports corroborate broad tunability: coarse, fully dense CoCrFeMnNi shows κ ~20–30 W·m^−1^·K^−1^ in some measurements, whereas purposely engineered nanostructured HEA coatings and dealloyed nanoporous HEA films reach κ < 1 W·m^−1^·K^−1^ due to pore scattering and amorphous-like regions [204,209]. These results point to an unusual design space where alloy chemistry, CSRO and microstructural features can be combined to target either high thermal transport (for heat-sink applications) or ultralow κ (for thermal barriers and thermoelectrics).

### 6.3. Corrosion and Oxidation Resistance

Corrosion resistance is a vital property for structural materials, especially in marine, offshore, chemical, and nuclear environments. HEAs show promise in this regard, often surpassing conventional stainless steels or nickel-based alloys in specific cases, due to the formation of stable, protective oxide layers and the absence of large, precipitated phases that might weaken the material. The coupling of mechanical and corrosion properties is a non-trivial issue. As described by Zheng, high mechanical strength may sometimes reduce corrosion resistance due to micro-galvanic coupling or micro-crack initiation sites [210].

Corrosion and high-temperature oxidation in HEAs depend sensitively on composition, the presence of passivating elements (Cr, Al, Si), and phase constitution. Recent work shows that Cr content, Al additions, and micro-alloying/second phase precipitates can significantly improve corrosion resistance in HEAs. The relative study found that increasing Cr content in porous FeCoNiMnCr_x_ reduced corrosion resistance due to increased porosity and permeability, indicating a nuanced interplay between composition, microstructure and corrosion [211].

In a study by Liu et al., it was shown that the addition of B in combination with Cr and Al improved both the corrosion resistance and high-temperature oxidation resistance of a CoCrFeNi-based HEA. These elements facilitated the formation of corrosion-resistant boride and oxide phases, particularly in saline environments [212].

Many equiatomic CoCrFeMnNi-type alloys show passivation behavior comparable to stainless steels in numerous environments, and oxide film formation kinetics can be slower due to the multicomponent surface chemistry that retards selective dissolution [213,214]. Numerical electrochemical metrics reported across studies vary with processing: for additively manufactured and cast CoCrFeNi variants, corrosion current densities (i_corr_) range broadly but improvements via minor Al or Cr enrichment and surface engineering have reduced i_corr_ by orders of magnitude in comparative tests [215]. For biomedical-oriented HEAs, several compositions have shown lower i_corr_ and higher open circuit potentials than Ti-6Al-4V under simulated body fluids, motivating interest in implant applications [216]. Biomedical HEAs often consist of equimolar or near-equimolar mixtures of biocompatible elements such as Ti, Nb, Ta, Zr, and Mo. For instance, the TiNbTaZrMo alloy has been extensively studied for its corrosion behavior in simulated body fluid (SBF) [217].

The specific SBF used in these studies typically follows the composition outlined by Kokubo, which mimics the ionic strength and pH of human blood plasma, providing a standardized medium for assessing the corrosion behavior of biomaterials. Electrochemical tests, including polarization resistance measurements, have demonstrated that this HEA exhibits a lower corrosion current density (i_corr_) compared to Ti-6Al-4V, indicating superior corrosion resistance in simulated physiological environments.

In addition, Roche et al. [218] investigated the passive film formation on the Ti_21_Nb_24_Mo_23_Hf_17_Ta_15_ HEA before and after immersion in SBF. Their results indicated that the alloy formed a stable and protective passive film upon immersion, leading to improved corrosion resistance. The authors attributed this enhancement to the alloy’s unique composition and microstructure, which facilitated the development of a robust passive layer.

At high temperatures, oxidation resistance depends on Al/Cr activity: refractory HEAs designed for Al-rich protective scale formation can show parabolic oxidation kinetics and stable Al_2_O_3_ scales rivaling Ni-based superalloys in some short-term tests, though long-term scale adherence and volatility remain open issues [5,219].

### 6.4. Catalytic and Electrocatalytic Activity

HEAs have emerged as a promising catalytic platform due to a vast combinatorial surface-site diversity that can present optimized adsorption energies for multistep reactions. Nanostructured HEA catalysts have shown outstanding activity for hydrogen evolution (HER), oxygen reduction (ORR), oxygen evolution (OER), CO_2_ reduction, and biomass oxidation. Representative electrochemical numbers: a dealloyed HEA OER surface showed an overpotential of ~370 mV at 40 mA·cm^−2^, while certain Ag–Ru–based HEA nanoparticles demonstrate bifunctional ORR/OER performance enabling fuel-cell currents and peak power densities above 100 mW·cm^−2^ in device tests [220,221]. For HER, some CoCrFeNi-derived HEA surfaces achieve onset potentials and current densities that approach benchmark nanostructured Pt-free catalysts (e.g., −0.32 V onset vs. −0.26 V for Pt at 1 mA·cm^−2^ in comparative lab tests) [222]. The large compositional space also allows tailoring of selectivity, exemplified by glycerol oxidation on a CoNiCuMnMo HEA reaching 10 mA·cm^−2^ at ~1.25 V vs. RHE with high product selectivity [223]. Challenges include scale-up, surface segregation under reaction conditions, and understanding active site distributions; recent reviews emphasize rational design via electronic-structure descriptors and high-throughput screening for accelerated discovery [224,225].

### 6.5. Magnetic and Spin-Related Phenomena

Magnetism in HEAs is richly varied: by compositional choice one can realize ferromagnetic (FM), ferrimagnetic, spin-glass, nearly paramagnetic and complex antiferromagnetic states. The high configurational disorder and possible local magnetic moment fluctuations generate anomalous coercivity, exchange bias and magnetocaloric responses in selected systems. Fe–Co–Ni–based HEAs retain significant saturation magnetizations while sometimes exhibiting reduced Curie temperatures relative to pure constituents, enabling tunable soft magnetic behavior [226]. Refractory and f-element containing HEAs also have been investigated for unusual spin textures and competing orders. Recent focused reviews summarize the field’s rapid expansion and quantify typical magnetization values, coercivities and phase-dependent magnetic anisotropies across families of HEAs [227].

### 6.6. Superconductivity

Superconductivity in HEA and medium-entropy alloys has become an active frontier: bulk, type-II superconductivity has been observed in several multicomponent systems (e.g., equiatomic TaNbZrTi(Hf) and certain hexagonal HEAs) with transition temperatures (T_c_) in the few-kelvin range. Very recent experiments report T_c_ ≈ 4.6 K in an equiatomic hexagonal HEA and other refractory HEA derivatives showing conventional s-wave pairing signatures in heat capacity and muon measurements [228,229]. Disorder-driven enhancement or suppression of superconductivity depends on electron count, phonon spectra and pair-breaking scattering—in some HEAs the high density of states and strong electron–phonon coupling counteract disorder to yield robust superconductivity, while in others magnetic impurities or strong spin–orbit scattering depress T_c_. The emergent possibility of topological superconducting states in carefully tuned compositional windows is under discussion and subject to active experimental pursuit [230].

### 6.7. Hydrogen Storage and Hydrogen-Interaction Phenomena

Hydrogen uptake and hydride formation in HEAs—particularly those containing volumetrically and chemically compatible elements such as Ti, V, Zr, Nb and Hf—have been widely studied. Early and later experimental studies show capacities spanning from ≲1 wt.% to reports of ~2–2.7 wt.% (≈2.5 H per metal in some refractory HEAs) under pressurized conditions and at elevated temperatures, with kinetics that vary strongly with phase (BCC vs. Laves vs. C14/C15 intermetallics) and microstructure [231,232]. For example, TiVZrNbHf alloys were reported to approach ~2.7 wt.% in some studies under hydrogenation conditions, while other chemically designed HEAs (Mg-containing BCC variants) have been reported with ~1.2 wt.% in specific tests [233,234]. Practical hydrogen-storage metrics (absorption/desorption pressures, cycling stability, heat management) remain challenging, but HEAs offer a route to combine fast kinetics (via catalytically active elements) and tunable thermodynamics.

### 6.8. Radiation Tolerance and Defect Physics

HEAs have attracted attention for radiation-tolerant materials because chemical complexity and sluggish diffusion are hypothesized to impede defect mobility and clustering. Ion-irradiation and neutron-irradiation experiments on canonical systems such as CoCrFeNi and CoCrFeMnNi show reduced swelling, suppressed void formation and enhanced retention of phase stability at fluences that produce significant damage in many single-component metals; for instance, sequential Kr and He irradiation up to fluences of 5 × 10^15^ cm^−2^ (Kr) and 2 × 10^17^ cm^−2^ (He) produced limited phase decomposition in CoCrFeNi and CoCrFeMnNi in one report [235].

A recent work [236] on WTaCrV HEA targeted for fusion reactor applications, shows that under helium-ion irradiation and cascade overlap up to ~0.2 dpa at 300 K, the HEA exhibited strong resistance to dislocation-loop formation and large interstitial clusters compared to pure W. However, the alloy was more susceptible to helium bubble formation. This indicates that HEAs with refractory content may offer improved defect tolerance under irradiation, which is a functional property of importance for nuclear/fusion applications [237]. Atomistic modeling implicates localized recombination and diverse trapping sites as mechanistic contributors, but quantifying long-term embrittlement and transmutation effects remains an open research priority.

### 6.9. Electronic Structure, Modeling and Design Principles

Predictive design of HEA functional properties leverages first-principles calculations, cluster expansion methods, machine learning, and high-throughput experiments. Electronic-structure calculations are now used routinely to screen candidate compositions for desirable densities of states, band alignments, and adsorption energetics for catalysis; cluster expansions and Monte-Carlo models capture CSRO effects that can crucially alter phonon spectra and electronic transport [3,238]. Data-driven frameworks integrated with thermodynamic databases and physics-based descriptors have begun to yield actionable composition–process–property maps for corrosion resistance, catalytic activity, hydrogen uptake and oxidation resistance [239,240]. Still, theoretical challenges remain: capturing strong disorder, electron–phonon coupling in multicomponent lattices, and long-range diffusion under nonequilibrium processing are active areas of method development.

### 6.10. Diffusion Coefficients and Transport Phenomena

Diffusivity in alloys influences creep behavior, phase stability, precipitation kinetics, oxidation penetration, and high-temperature performance. HEAs have been proposed to exhibit “sluggish diffusion”, though this concept has been debated. A key recent paper [241] used tracer diffusion techniques to quantify grain-boundary diffusion paths in equal-channel angular pressing processed HEA. Researchers found three distinct diffusion paths associated with twin boundaries, high-angle grain boundaries, and porosity/defect networks. The measured enhancement of diffusivity was attributed to high strain localization near interfaces and deformation-induced non-equilibrium states. Although the absolute numerical diffusion coefficients were not always directly comparable to classic alloys, the study emphasizes that micro-structural engineering (grain size, deformation state) strongly affects diffusivity in HEAs.

The chemical potentials of vacancy and interstitial formation in the FCC CoCrFeMnNi HEA were quantified by Gao et al. [242]. They reported the chemical potential values for each constituent element for vacancy/interstitial formation, indicating variable energetics across elements in the HEA matrix. For example, μ_Cr_ in the HEA was larger than in pure Cr, and μ_Mn_ was smaller than pure Mn, reflecting the altered defect energetics in the multicomponent environment. This suggests that diffusion and defect mobility in HEAs is compositionally dependent and cannot be approximated by simple “sluggish diffusion” across the board.

Although definitive diffusion coefficient values for a large number of HEAs are not yet collated, the emerging pattern suggests that HEAs processed to fine grain size or with high defect density may actually exhibit higher diffusivity along defects/grain boundaries than coarse-grained HEAs. However, the bulk lattice diffusion may still be lower than in simple alloys, especially when interstitials or refractory elements are added which raise defect formation energies and reduce atomic mobility.

### 6.11. Stacking Fault Energy (SFE), Deformation Mechanisms and Phase Stability

Stacking fault energy (SFE) is a crucial parameter controlling plastic deformation modes. Low SFE leads to twinning/ε martensite formation, while high SFE is linked to dislocation glide/slip bands. For HEAs, the ability to tune SFE via composition or interstitial addition is a powerful design lever. The recent work of [243] reports that for equimolar FeCoNiCrMn HEA the calculated SFE is in the range of −93 to −5 mJ m^−2^ at 0 K, suggesting a propensity for martensitic transformation at low temperature. The same review notes that doping hydrogen decreases SFE (making twinning/MT more likely), while doping carbon or nitrogen increases SFE (thus favoring dislocation slip). Although many SFE values remain modeled rather than measured, the work by Shih et al. [244] indicates that the HEA’s heterogeneous atomic environment leads to spatial variability of SFE and non-uniform slip/twinning behavior.

### 6.12. Thermal Expansion in High Entropy Alloys

Thermal expansion in HEAs emerges as a deeply composition- and microstructure-dependent functional property whose control is critical for integration into precision, high-temperature, and thermomechanically demanding applications. The large configurational complexity and associated lattice distortion in multicomponent HEAs decouple coefficient of thermal expansion (CTE) from simple rule-of-mixtures expectations and instead produce a broad, tunable spectrum of thermal response through effects on bonding character, phonon spectra and defect populations [119].

Recent theoretical and experimental work shows that lattice anharmonicity—amplified by chemical disorder, atomic size mismatch and local chemical short-range order—is a primary driver of non-trivial temperature dependence of lattice parameters in HEAs and therefore must be treated beyond the harmonic approximation to capture CTE accurately [245].

First-principles and atomistic modeling (quasi-harmonic DFT, special quasirandom structures and molecular dynamics) have proven capable of predicting CTE trends in refractory BCC HEAs (e.g., Mo–Nb–Ta–Ti–W systems), providing design rules that link element selection and electronic/elastic response to measurable thermal expansion [246].

Experimentally, HEAs display an unexpectedly wide palette of behaviors—from near-zero and suppressed CTEs in carefully engineered multiphase and precipitation-hardened alloys to reports of compositionally complex alloys exhibiting pronounced negative thermal expansion (NTE) over extended temperature windows—demonstrating that compositional complexity can be harnessed to invert or flatten thermal response when coupled to appropriate phase chemistry or lattice-dynamical mechanisms [247].

For example, the addition of Zr and Si to a base Al_0.5_CoCrFeNi HEA suppressed the CTE, reducing thermal mismatch in multilayer heater coatings—highlighting how purposeful compositional tuning can tailor expansion behavior for application-specific thermal resilience [248]. Moreover, the experimental demonstration of nearly zero or even negative thermal expansion (NTE) over a wide temperature range in a high-entropy magnet compound points to further opportunities: coupling magneto-structural transitions and configurational disorder to control volumetric dilation [249].

Building on this, recent materials-by-design strategies exploit in situ formation of nanoscale negative-expansion phases, lamellar architectures, or controlled precipitation to achieve macroscopically reduced or tailored CTE while preserving mechanical integrity at elevated temperatures. This approach directly addresses the long-standing mismatch problem in precision structures and high-temperature functional devices [250].

Practically, these advances imply that HEA design for thermal management must co-optimize electronic structure, elastic anisotropy, phase stability and defect energetics. CTE cannot be treated in isolation, but as an emergent thermodynamic quantity tied to phase equilibria and anharmonic phonon lifetimes, and its reliable control will increasingly rely on integrated high-throughput computation, targeted alloying strategies, and microstructural engineering validated by in situ high-temperature metrology [245].

Therefore, in HEAs, CTE must be understood not merely as a scalar material constant but as a complex emergent property arising from interwoven atomic-scale mechanisms-making it a pivotal parameter in assessing their viability for functional applications, especially under thermomechanical cycling or across temperature gradients. Recent theoretical work supports that thermal expansion in HEAs is strongly influenced by (i) the magnitude of static lattice strain generated by atomic size mismatch and chemical disorder, which elevates the phonon-anharmonicity and thus increases the intrinsic volumetric expansion; (ii) the configurational entropy stabilization of single-phase solid solutions, which suppresses phase transformations that might otherwise alter the thermal expansion behavior; (iii) the coupling between lattice vibrations (phonons) and local electronic/defect states, especially in multicomponent systems where mass and force-constant disorder broaden phonon density of states and reduce thermal conductivity—consequently modifying thermal expansion via Grüneisen parameter variations (as recently modeled for the CoNiFe system) [251].

From a design perspective, these findings imply that achieving a target thermal expansion entails balancing three levers: alloy selection (to control lattice distortion and interatomic stiffness), microstructural stability (to avoid phase decomposition that alters volumetric response with temperature, as in refractory systems above ~1000 °C) [252] and phonon engineering (via mass contrast, defect scattering and electronic coupling) to modulate the effective Grüneisen parameters. The strong compositional and microstructural sensitivity of CTE in HEAs thus transforms it from a fixed material constant into a tunable output of alloy design, making it a cornerstone criterion in evaluating their real-world applicability for functional applications, where dimensional stability, thermal cycling and compatibility with adjoining materials (e.g., ceramics, coatings, precision structures) are critical.

### 6.13. Grain Boundaries and Functional Properties of High-Entropy Alloys

Grain boundaries (GBs) in HEAs act as chemically and structurally distinct interfacial reservoirs whose local thermodynamics and kinetics exert outsized control on macroscopic functional properties and microstructural stability. Recent high-resolution experiments and atomistic modeling collectively show that GBs in FCC HEAs (for example CoCrFeMnNi and derivatives) are rarely compositionally uniform. Specific species (notably Cr, Mn and, in some heat-treatment conditions, Ni) can show boundary-type and temperature-dependent enrichment or depletion, producing compositional gradients, short-range order (SRO) and nanoscale clustering at interfaces that alter cohesive strength and the criteria for dislocation emission from GBs [253].

Thermodynamically, the notion of “high-entropy grain boundaries” (HEGBs) has been formulated and quantified: configurational entropy associated with occupation of multiple species on GB sites can reduce the effective GB free energy and modify the balance between capillarity and solute drag, thereby retarding curvature-driven grain growth and stabilizing nanocrystalline or ultrafine grain structures at elevated temperatures. This thermodynamic picture—supported by theoretical models and targeted experiments—predicts that the effective GB entropy and segregation free energy vary with the number of principal elements, temperature and the degree of GB saturation, making GB states in HEAs inherently state-dependent rather than fixed [112,254].

Mechanistically, GB segregation plays dual and sometimes competing roles. Beneficial outcomes arise when segregants pin dislocations, increase the stress required for dislocation nucleation at GBs, or form thin structurally disordered (or amorphous) intergranular films that blunt crack tips and increase ductility. Conversely, detrimental outcomes occur when co-segregation or impurity enrichment produces embrittling intergranular phases, SRO-driven nanoclusters that locally reduce cohesion, or chemical environments that accelerate intergranular corrosion. Several correlative studies using atom probe tomography (APT) together with electron microscopy now document both strengthening and embrittling scenarios in HEAs, demonstrating the sensitivity of interfacial response to small changes in bulk composition and thermal history [255].

Interfacial complexions—thermodynamically distinct GB structural/chemical states including ordered, disordered and amorphous intergranular films—have been detected or inferred in HEAs and are now recognized as an important control knob for functional properties. Amorphous intergranular films (AIFs) and other complexion types can lower GB diffusivity and suppress grain growth in some chemistries but can also act as fast diffusion pathways or sites for oxidation/attack under differing environmental chemistries. Thus, complexion identity and stability must be considered together with segregation chemistry and external chemical potential (oxygen, S, etc.) [256].

On the computational side, advances in atomistic simulation (MD/Monte-Carlo hybrids), thermodynamic segregation models, and machine-learning-assisted descriptors increasingly permit predictive mapping between bulk composition/processing and likely GB states. These frameworks can forecast segregation tendencies, complexion transitions, and the sensitivity of GB energy and mobility to chemistry and temperature. These approaches enable strategy-oriented grain-boundary engineering via minor-element doping, targeted heat treatments, or process routes that favor beneficial GB chemistries. When combined with experimental efforts and correlative microscopy, these models can produce a shift from descriptive observations toward prescriptive design rules for GBs in HEAs [72,257].

Finally, experimental capability advances-notably methods for quantifying SRO using APT and correlative microscopy that link atomic-scale chemistry to mesoscopic mechanical response—are sharpening mechanistic understanding and opening practical pathways for stabilizing beneficial GB states in HEAs. Together, these developments support a paradigm where GB engineering (controlled segregation, complexion selection, and thermal-mechanical processing) becomes an explicit element of HEA design for tailored mechanical, thermal and environmental performance [258].

### 6.14. Outlook and Unresolved Challenges

HEAs offer an unusually broad functional design space, but practical deployment hinges on addressing stability under service conditions (segregation, selective oxidation, loss of active sites), scalable synthesis of nanostructured and porous morphologies, and improved predictive models linking local atomic arrangements to macroscopic functional metrics. Cross-disciplinary efforts combining high-throughput synthesis, operando spectroscopy, and multiscale modeling are accelerating discovery; however, establishing standardized test protocols (particularly for corrosion, electrocatalysis and hydrogen storage metrics) would greatly improve comparability across studies. Continued emphasis on linking measurable numerical benchmarks (i_corr_, overpotentials at fixed current densities, hydrogen wt.% under defined P/T, thermal conductivity at 300 K, T_c_ for superconductors) to design rules will be essential for translating HEA functional promise into real applications.

Furthermore, the economic drivers and barriers for deploying HEAs in functional roles should be further discussed. In general, the economic viability of HEAs targeted for functional applications depends on three coupled factors: (1) raw-material composition and commodity price exposure, (2) processing and fabrication costs (including powder production and advanced routes such as additive manufacturing), and (3) use-phase value added (performance benefits that permit downsizing, lifetime extension, energy savings or novel functionality). Quantitative appraisal of these factors shows that HEAs can be cost-competitive for niche, high-value functional uses, but face clear barriers for bulk replacement of commodity engineering alloys.

To justify the raw and processing premium, a HEA must deliver one of the following measurable economic outcomes in the application context:

- Mass or volume reduction, e.g., if a HEA coating or thin structural element halves component mass while retaining functionality, the net system cost may fall when system-level costs (fuel, inertia, installation) dominate.
- Extended service life: in offshore, chemical or high-temperature environments a 3–5× extension in replacement interval converts into strong net present value (NPV) gains even when material unit cost is higher.
- Energy efficiency gains: functional HEAs used in thermoelectric or catalytic roles that improve conversion efficiency by a few percentage points can pay back material costs over device lifetimes if the energy value is high (grid-scale or industrial heat recovery).
- Capability enabling: certain HEA combinations enable functionalities (e.g., simultaneous corrosion resistance and high saturation magnetization) not achievable with conventional alloys; such unique capabilities permit premium pricing analogous to specialty ceramics or rare magnets.

Industry market estimations consistently place the nascent HEA market in the low-single-digit billion USD range by the 2030s, with Compound Annual Growth Rate (CAGR) projections typically in the 7–17% range depending on source and segmentation. These market sizes indicate a currently small but rapidly growing domain, suitable for high-value sectors (aerospace, defense, energy conversion devices) rather than mass automotive or construction use. Early commercial traction is most plausible where HEAs enable functional advantages that translate directly to lifecycle economic benefit (e.g., thermoelectric modules with higher ZT leading to measurable fuel savings; magnetic refrigeration materials with higher cooling power density enabling smaller systems; corrosion-resistant electrical contacts reducing replacement frequency) [259].

To conclude, techno-economic assessments for HEA functional uses should (i) employ up-to-date spot prices for constituent metals in a sensitivity envelope (±30–50%), (ii) model fabrication cost multipliers specific to the selected route (casting vs. PM vs. AM) and (iii) include recycling scenarios and policy-risk scenarios.

## 7. Applications and Future Perspectives

### 7.1. Introduction

High-entropy alloys (HEAs), first brought to prominence by Yeh and co-workers and independently explored by Cantor’s group in 2004, opened a paradigm in alloy design by moving away from single-principal-element metallurgy toward near-equiatomic multi-principal-element alloys; this conceptual shift—together with the four “core effects” (high mixing entropy, severe lattice distortion, sluggish diffusion and cocktail effects)—has driven an explosion of both fundamental studies and application-oriented research over the past two decades [141,142]. Early foundational and critical assessments established both the promise and the realistic constraints of HEAs, providing a map for application areas where their combination of mechanical performance, thermal stability, corrosion resistance and multifunctionality might outperform conventional alloys [146,147,260,261].

### 7.2. Structural and Load-Bearing Applications (Aerospace, Automotive, Tooling)

HEAs have been extensively explored as structural materials for demanding load-bearing applications due to their exceptional combination of strength and ductility in select compositions. The prototypical medium-entropy CrMnFeCoNi Cantor alloy family exhibits tensile strengths on the order of ~1 GPa with ductilities often reported in the tens of percent (e.g., 50–70% elongation in optimized processing routes), and outstanding fracture-toughness metrics that make them competitive with conventional stainless steels in damage-tolerant applications [262].

Refractory HEAs (RHEAs) such as designed Nb–Mo–Ta–W and newer natural-mixing refractory compositions show promise for very high temperature structural use. These compositions are designed to exploit high mixing entropy, which promotes the formation of single-phase solid solutions, thereby enhancing high-temperature stability and mechanical properties. Indicative examples of natural-mixing refractory HEAs are the Ti_3_Zr_1.5_NbV_x_ alloys which are designed to achieve a balance between strength and ductility at high temperatures. The incorporation of vanadium in varying amounts allows for tuning the mechanical properties, making them suitable for applications requiring both high strength and reasonable ductility [263].

Additionally, HfNbTaTiZr (equiatomic) alloy exhibits good room-temperature ductility, with up to 50% deformation achievable under compression. However, its high-temperature yield strength is relatively low, indicating a trade-off between ductility and strength at elevated temperatures [264].

Recent designs have demonstrated as-cast tensile ductilities exceeding 20% while retaining very high yield strengths at elevated temperatures, a class of behavior uncommon for traditional refractory alloys [119,265]. These numerical examples illustrate that HEAs can traverse the traditional strength–ductility tradeoff by exploiting complex, synergistic deformation mechanisms (partial dislocation activity, nano-twinning and sustained work hardening) and by microstructural engineering (precipitates, heterogeneous grain structures). Nevertheless, scale-up and cost (refractory element content) remain practical hurdles for large structural components, so near-term adoption is likely to focus on niche, high-value aerospace and tooling components where performance justifies cost.

### 7.3. High-Temperature and Refractory Uses (Power Generation, Turbines)

For ultra-high temperature environments (gas turbines, concentrated solar receivers and advanced rocket components), RHEAs were proposed because of their very high melting points and sluggish softening at temperature. Systematic studies over the past five years have quantified creep and oxidation responses of model RHEAs, with several compositions demonstrating superior creep-resistance relative to some Ni-based superalloys at temperature ranges where oxide scale formation and elemental volatility are limiting in conventional alloys; for example, carefully tuned W- and Ta-bearing RHEAs can maintain creep lifetimes and yield strengths at temperatures above 800–1000 °C that rival or exceed conventional alloys in short-term tests [5,120]. However, oxidation resistance, environmental embrittlement and manufacturability (castability, weldability) remain active research problems; surface engineering and alloying strategies (Al/Ti additions to form protective alumina/scale layers, controlled oxide dispersion) are being developed to close gaps to commercial superalloys.

### 7.4. Wear, Tribology and Protective Coatings

HEA coatings and surface treatments have rapidly become a major application thrust because depositing a thin, HEA-derived layer can impart combined hardness, toughness and oxidation/corrosion resistance while minimizing the material cost of bulk refractory HEAs. Laser cladding, thermal spraying, magnetron sputtering and emerging additive deposition routes have produced HEA coatings with hardness increases of 20–200% and significant reductions in wear coefficients compared to baseline steels or Ni-based coatings in laboratory sliding and abrasive tests; in several comparative studies, multi-component coatings outperformed conventional single-phase hard coatings especially under complex loading and corrosive wear conditions [119,266]. Additive manufacturing (AM) routes—laser powder bed fusion and directed energy deposition—enable near-net-shape deposition of HEA coatings and graded transitions to substrate materials, which reduces interfacial failures and enables tailored tribological layers for tooling and forming dies [120,265].

### 7.5. Energy, Corrosion Resistance and Electrochemical Applications

HEAs have attracted strong interest in energy conversion and storage because their multi-element surfaces offer rich active sites and corrosion-tolerant matrices. Studies show HEA electrodes and catalysts can deliver improved stability and activity for oxygen evolution/reduction, hydrogen evolution and CO_2_ reduction reactions; for instance, recent HEA electrode materials show competitive current densities and markedly improved operational lifetimes under harsh electrochemical conditions compared to benchmark noble-metal electrodes [267,268].

In more details, HEA electrodes have been demonstrated across the community for the principal electrocatalytic reactions relevant to energy conversion—notably the hydrogen evolution reaction (HER), oxygen evolution reaction (OER), and oxygen reduction reaction (ORR) and, increasingly, CO_2_RR and nitrate/other small-molecule reductions—rather than a single reaction class. In terms of activity, many HEA systems achieve current-density benchmarks comparable to or better than conventional noble-metal catalysts. Examples include HEA nanosheets that reach 10 mA·cm^−2^ at very low overpotentials (e.g., ≈13 mV for a Pd–Mo–Ga–In–Ni HEA vs. commercial Pt/C) and HEA electrodes achieving 10 mA·cm^−2^ at overpotentials on the order of 0.19–0.37 V for overall water splitting/HER–OER couples, i.e., performance in the same ballpark as Pt- or Ir-based materials [269].

In terms of durability, HEA catalysts frequently show markedly improved operational lifetimes. Stability tests reported in literature span from tens of hours to well over 1000 h depending on composition and testing current density. Representative examples are HEA cathodes that operate stably for ~200 h at 100 mA·cm^−2^ (minimal Pd leaching compared with Pd/C), chronopotentiometry/chronoamperometry tests showing stable HER at −500 to −1000 mA·cm^−2^ for ~100 h, and recent HEA anodes/LOM-active HEAs demonstrating >1000–1500 h of continuous operation at practical current densities. In many head-to-head comparisons the reported activity loss is small (often <10–20% over ten-to-hundreds of hours), which in several cases represents an order-of-magnitude improvement in lifetime compared with co-tested nanoparticle catalysts that show rapid aggregation or metal leaching under the same conditions.

Mechanistically, these gains are attributed to the multielement “cocktail” effects (tunable binding energies and many adjacent active motifs), sluggish diffusion/reduced coarsening, and surface-oxide/coordination environments that suppress dissolution [270].

In corrosion studies, many HEAs (especially Cr-, Al-, or Ni-containing families) also exhibit passivation and pitting resistance comparable to stainless steels, with reports of substantially reduced corrosion rates in chloride media for optimized compositions. The combination of catalytic multifunctionality and corrosion resistance makes HEAs attractive for electrolyzers, fuel-cells and seawater-resistant components, though precise control of surface segregation and oxide formation is required to harness catalytic active sites reproducibly [267,271].

### 7.6. Catalysis, Nanoparticles and Functional Surface Materials

High-entropy alloy nanoparticles (HEA-NPs) constitute a rapidly growing subfield in which the same “cocktail” effect that stabilizes bulk solid solutions is exploited at the nanoscale to create multicomponent active sites with tunable ensemble geometries. Reviews and experimental demonstrations report HEA-NP catalysts with broad adsorption energy distributions that can break conventional scaling relationships and achieve notable improvements in selectivity and stability for complex reactions (e.g., multi-electron CO_2_-to-C^2+^ pathways). Quantitatively, recent HEA-NP studies have reported turnover frequency (TOF) improvements of factors of 2–10 over monometallic catalysts in specific reactions and significantly lower deactivation rates in long-duration tests, illustrating potential commercial relevance, especially where multi-function catalysts are needed [267,272]. Synthesis methods (carbothermal shock, laser ablation, wet chemical routes) and characterization campaigns (operando spectroscopy, atom-probe tomography) are rapidly converging to enable rational HEA nanoparticle design.

### 7.7. Additive Manufacturing, Joining and Component Integration

Additive manufacturing (AM) is perhaps the most practical near-term industrial enabler for HEAs because AM tolerates compositional flexibility, enables complex geometries, and can produce functionally graded or lattice architectures that exploit HEA behavior. Recent AM-HEA work has mapped processing windows (laser power, scan speed, hatch spacing) to microstructural outcomes, showing that AM routes can produce fine cellular solid solutions, controlled heterogeneities, and feature sizes that improve combinations of strength and toughness; specific studies report densified AM parts with tensile strengths and ductilities comparable to wrought HEA material, though residual stress, porosity and evaporation of volatile elements need careful mitigation [262,263]. Wire-arc AM and directed energy deposition expand the scale of HEA components for large parts, but require powder/wire feedstock optimization and post-build heat-treatments to control segregation and phases [271]. Thus, AM combined with HEA design opens pathways to application in tooling, bespoke aero-components and multifunctional structural parts.

### 7.8. Biomedical and Implantable Uses

Interest in HEAs for biomedical implants stems from the ability to tailor elastic modulus, corrosion resistance and surface chemistry by composition and thermomechanical processing. Some HEA formulations (e.g., Ti-containing HEAs) have shown biocompatibility and osseointegration potential with corrosion resistance under simulated body fluids, while low-modulus HEAs have been explored to reduce stress-shielding in load-bearing implants. Nevertheless, stringent biocompatibility testing, ion-release profiles and long-term in vivo studies are limited and required before translation; regulatory pathways are nontrivial for multicomponent alloys with less historical use. Early numerical/live-animal examples are promising but the field is still at the materials-development stage rather than clinical readiness [141,273].

### 7.9. Radiation Resistance and Nuclear Applications

Because sluggish diffusion and complex local chemistry can suppress defect mobility, several HEAs have demonstrated improved radiation resistance (reduced swelling, reduced defect cluster growth) in ion-irradiation and neutron-irradiation experiments relative to benchmark steels and Ni alloys. For example, irradiated concentrated alloys based on stainless-steel-like chemistries show delayed void formation and retained mechanical performance at high doses, making HEAs attractive for next-generation reactors and fusion components where radiation tolerance and thermal stability must coexist. Still, data for long-term neutron fluences, weldability in reactor-grade components and helium-induced embrittlement are areas of ongoing, required study before licensing for nuclear structural use [141,146].

### 7.10. Functional Applications—Magnetic, Electronic, and EMI Shielding

Beyond structural and catalytic roles, HEAs are being investigated for functional properties: tunable magnetic behavior (from soft to hard magnetism by composition), thermoelectric candidates, and electromagnetic interference (EMI) shielding materials that leverage multi-phase microstructures and conductive/ferromagnetic constituents. Recent reviews highlight high-entropy metallic glasses and composites as promising for EMI shielding with favorable specific shielding effectiveness and corrosion tolerance compared to polymer-based composites; these functional uses are attractive for electronics in harsh environments and for integrated structural-functional components [260].

### 7.11. Future Perspectives and Research Directions

The future of HEAs will be driven by a convergence of computational design, high-throughput experiments, machine learning (ML), and advanced processing (AM, coatings, nanoparticle synthesis). Machine-learning frameworks have already accelerated discovery by screening vast compositional spaces and predicting phase formation, hardness and oxidation tendencies; several algorithmic studies have successfully suggested candidate compositions with targeted hardness increases or phase stability and subsequent experimental validation (e.g., algorithmic optimization yielding ultra-hard compositions with predictive accuracy within experimental uncertainty) [73,259]. High-throughput combinatorial fabrication (thin films, micro-samples) coupled with automated characterization and ML-driven active learning loops promises to reduce discovery time from years to months for targeted application windows [261,265].

Replacing or minimizing critical or scarce elements (Re, W, Ta) via “economical HEA” design, using recycling-friendly element sets, and developing graded architectures that use HEA only where needed (surface layers, inserts) will be essential. In parallel, lifecycle assessment, joining/welding standards for multi-principal-element alloys, and industrial-scale powder/wire supply chains must be established; such infrastructure work is equally important as discovery. Computational thermodynamics (CALPHAD extensions) and atomistic methods must be further adapted to handle truly high-dimensional composition spaces for reliable phase and property prediction [3,274,275].

Another exciting trajectory is the expansion of the “high-entropy” concept to ceramics, oxides, nitrides and thin films: high-entropy ceramics and oxides open possibilities for functional ceramics with tunable ionic conductivity, thermal barrier coatings, and multi-functional electronic ceramics; these materials inherit the configurational complexity but bring distinct defect chemistries that can be targeted for sensors and energy devices. HEA derivatives in nanoscale and interstitially stabilized forms (oxygen-complex strengthened HEAs) have already shown new deformation and hardening modes, implying that hybrid design strategies (multiphase + interstitial engineering) will produce application-ready materials that outperform single-phase analogs [273,275].

### 7.12. Performance Metrics and Economic Considerations Regarding HEAs Applications

High-entropy alloys (HEAs) exhibit a suite of performance metrics that span exceptional mechanical strength and promising, though still variable, electrocatalytic activity; quantitatively, recent nanoscale HEA electrocatalysts such as FeCoNiMnRu/CNFs have delivered hydrogen- and oxygen-reaction overpotentials of ≈71 mV (HER) and ≈308 mV (OER) at 100 mA·cm^−2^, demonstrating that carefully tuned multicomponent surfaces can approach the activity of precious-metal benchmarks in practical current regimes [276].

On the contrary, state-of-the-art Pt-based materials still set the low-overpotential standard (e.g., Pt–Ru and ultralow-Pt formulations report overpotentials as low as ≈18–45 mV at 10 mA·cm^−2^ for HER in alkaline media), so HEAs are best presently viewed as either Pt-sparing supplements or as routes to improved stability/poison-tolerance rather than universal replacements [277].

Regarding the mechanical properties, HEAs targeted to aerospace applications (lightweight and refractory families) already show competitive specific-strength figures: several Al- and refractory-based HEAs report specific strengths on the order of 50–200 MPa·cm^3^·g^−1^ at elevated temperatures and tensile strengths in the several hundred to >1000 MPa range depending on composition and processing, which places them in the performance envelope of Ti- and Ni-based structural alloys while offering superior high-temperature retention in some cases [278].

From an industrial and economic perspective, however, the promise of HEAs is tempered by processing costs and scale-up complexity: additive manufacturing (AM) routes (PBF, DED, WAAM) enable near-net shaping and rapid alloy exploration but incur higher feedstock (pre-alloyed or gas-atomized powder) costs, larger unit processing and post-processing expenses, and sensitivity to defect formation that raises qualification costs relative to mature casting/wrought routes; techno-economic and cost-modeling studies therefore show that AM is most competitive for low-volume, high-value HEA components (e.g., bespoke aerospace parts) and that lifecycle/process optimization (feedstock sourcing, powder reuse, reduced post-processing) is required to close the cost gap for broader adoption [279].

Taken together, these metrics indicate that HEAs already offer compelling property gains (specific strength, thermal stability, and tunable surface chemistries) backed by accelerating discovery workflows, but their industrial impact will depend critically on (i) continuing reductions in AM and powder-production costs, (ii) head-to-head durability and mass-activity benchmarking against optimized Pt formulations across relevant current densities, and (iii) demonstration of repeatable, defect-controlled AM processing at production scale [280].

### 7.13. Concluding Remarks

High-entropy alloys represent a versatile materials platform with demonstrable achievements in structural toughness, high-temperature strength, corrosion resistance and catalytic multifunctionality. The next decade will determine whether HEAs transition from high-profile academic prospects to engineered solutions: that transition depends on integrating predictive computational tools, scalable manufacturing (especially AM), economical alloy formulations and rigorous environmental/regulatory validation. If these pieces come together, HEAs and their derivatives are poised to deliver disruptive materials solutions across energy, aerospace, catalysis and electronics.

## 8. Challenges and Opportunities

### 8.1. Cost, Sustainability, and Industrial Viability

Despite their scientific appeal, high-entropy alloys (HEAs) face a fundamental barrier when moving from laboratory discovery to industrial deployment: cost and resource sustainability. Many high-performance alloys, particularly refractory HEAs such as NbMoTaW or VNbMoTaW, rely on elements that are both expensive and scarce, such as Nb, Ta, and W [281]. The price volatility of these elements, coupled with their concentration in limited geographic regions, raises concerns about long-term supply security and geopolitical risk. For example, the raw-material cost of NbMoTaW has been estimated to be more than five times that of conventional Ni-based superalloys when calculated on a per-kilogram basis [282]. This economic limitation constrains HEAs primarily to niche, high-value applications where superior performance justifies investment, such as aerospace turbine components or advanced nuclear systems.

Sustainability considerations further complicate the industrial outlook. Recycling conventional steels and Ni-based alloys is already challenging; recycling HEAs with four or five principal elements is considerably more complex [283]. The thermodynamic driving forces that stabilize HEAs in use become a liability during recycling, as separating constituent elements for reuse is extremely energy intensive. Recent life-cycle analyses have emphasized the importance of designing “green” HEAs, which either minimize critical elements or employ more abundant substitutions (e.g., replacing Ta or W with Fe, Mn, or Al) [284]. Emerging strategies include the use of high-entropy medium-cost alloys (HEMCAs), where compositions are deliberately tuned to reduce reliance on expensive refractory elements while preserving mechanical and thermal stability [285,286].

Industrial viability also depends on process scalability. Additive manufacturing, while promising for producing HEAs in complex geometries, remains costly for large-scale production and requires strict powder control [143,287]. Conventional casting routes encounter segregation issues that increase scrap rates and reduce yield [288]. Powder metallurgy is more precise but is energy intensive and capital expensive [289]. Industrial adoption will therefore require optimized processing–cost balance, possibly leveraging hybrid routes such as casting followed by thermomechanical treatment or targeted AM for high-value components.

### 8.2. Future Directions and Integrative Strategies

**Figure 6:**
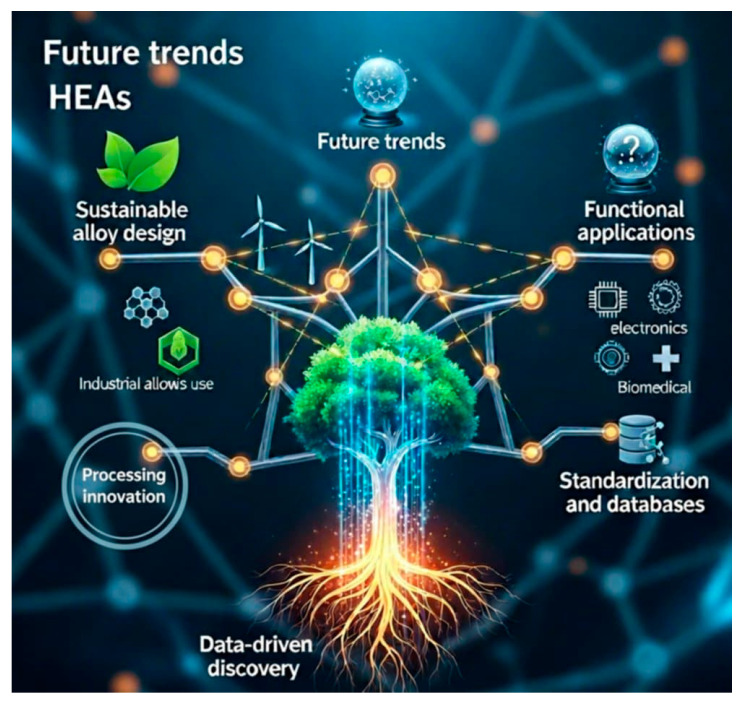
Schematic illustration of future trends in high-entropy alloys, highlighting advances in crucial fields of research and innovation.

The trajectory of HEA research is shifting from exploratory discovery toward application-oriented design. Several converging trends define the most likely future directions (Figure 6):

(i) Data-driven discovery. Machine learning (ML) and high-throughput computational frameworks are rapidly maturing and now play a central role in narrowing the HEA compositional space [290]. By integrating CALPHAD-based thermodynamics with ab initio calculations and ML predictors, researchers have begun to propose design frameworks that can screen thousands of candidate alloys in days, reducing reliance on costly trial-and-error experiments [291].
(ii) Processing innovation. Improving process control is critical to industrial adoption. AM routes are expected to mature, with better control of powder composition and melt pools, while hybrid approaches combining AM and heat treatments are being explored to enhance microstructural uniformity [292]. Powder metallurgy may benefit from sustainable feedstock strategies, such as recycling industrial scrap into HEA powders [293,294].
(iii) Sustainable alloy design. Next-generation HEAs will increasingly incorporate sustainability as a design criterion. Instead of optimizing purely for performance, alloys will be engineered for recyclability, low environmental footprint, and reduced reliance on critical elements [295]. For example, Fe-based HEAs enriched with Mn, Al, and Cr have been identified as low-cost alternatives for corrosion-resistant applications [296].
(iv) Functional applications. While structural alloys dominate current HEA research, functional HEAs in catalysis [297], energy storage [298], superconductivity [299], and EMI shielding [300] are emerging rapidly. These applications often require only small quantities of material, which alleviates cost pressures and accelerates early commercialization.
(v) Standardization and databases. The absence of standardized datasets remains a major bottleneck [301]. For example, a serious limitation in applying machine learning to high-entropy alloy design is the absence of standardized datasets encompassing critical material descriptors. Phase stability information-identifying single-phase versus multiphase structures (FCC, BCC, HCP) with explicit temperature and processing context-is often inconsistently reported, undermining predictive accuracy. Similarly, precise elemental compositions, including minor impurities, are rarely uniformly documented, complicating model generalization. Thermodynamic descriptors such as mixing enthalpy, entropy, valence electron concentration, and atomic size mismatch are frequently calculated with differing conventions, further fragmenting data integration. Processing history-including synthesis methods, cooling rates, and annealing protocols-is usually omitted or inconsistently described, despite its strong influence on microstructure and properties. Finally, mechanical and functional properties, such as tensile strength, hardness, and corrosion resistance, are measured under varying conditions, reducing the reliability of cross-study comparisons. Collectively, the lack of standardized reporting in these parameters represents a critical bottleneck for robust, generalizable machine learning models in HEA research.

### 8.3. Concluding Perspective

The cost and sustainability challenges of HEAs highlight a key reality: not all promising laboratory compositions will reach industrial practice. However, the unique mechanical and functional properties observed in carefully engineered HEAs justify continued exploration, particularly for sectors where extreme conditions or multifunctionality demand more than conventional alloys can deliver. By aligning alloy design with sustainability principles, integrating advanced computation and manufacturing, and targeting application-driven niches, HEAs have the potential to evolve from a scientific curiosity into a transformative class of materials in the coming decades.

## Figures and Tables

**Table 1:** Summarizes the strengths, limitations, and typical outputs of each of these computational methods, applied to HEAs. Future progress will depend on coordinated efforts to develop benchmark datasets, open databases, and community-wide platforms for reproducible, uncertainty-aware computational workflows [4]. The synergy between physics-based simulations and data-driven methods promises a new era of accelerated HEA discovery, design, and deployment.

Method	Strengths	Limitations	Typical Outputs
**Density Functional Theory (DFT)**	- First-principles accuracy (electronic structure level) - Predicts phase stability, defect energetics, electronic/magnetic properties - No need for empirical parameters	- Computationally expensive for large/highly disordered systems - Limited to small cells and short timescales - Approximations in exchange-correlation functionals	- Formation enthalpies - Phase stability maps - Elastic constants - Electronic density of states (DOS), band structure
**CALPHAD (CALculation of PHAse Diagrams)**	- Efficient for multi-component alloys - Can handle experimental + theoretical data - Provides thermodynamic modeling for high-order systems	- Accuracy depends on thermodynamic databases - Limited predictive power for unexplored compositions - May oversimplify configurational/atomic-scale effects	- Phase diagrams (T-x, T-P) - Gibbs free energies - Solidification pathways - Phase fractions vs. T
**Cluster Expansion + Monte Carlo (CE + MC)**	- Captures configurational entropy and chemical ordering - Efficient for predicting phase stability in disordered alloys - Scales better than pure DFT for large systems	- Requires accurate DFT training data - Limited to substitutional disorder (mainly solid solutions) - Computationally intensive for very high-order systems	- Configurational phase diagrams - Order–disorder transition temperatures - Short-range order parameters
**Molecular Dynamics (MD)**	- Captures finite-T and dynamical effects (diffusion, mechanical behavior) - Can simulate microstructural evolution - Suitable for large systems (105–106 atoms)	- Accuracy depends on interatomic potentials - Limited simulation timescales (ns–μs) - May miss rare events and long-term stability	- Diffusion coefficients - Mechanical properties (stress–strain curves, yield strength) - Thermal conductivity - Defect evolution
**Machine Learning (ML)**	- Fast screening of large compositional spaces - Can uncover hidden correlations in multi-dimensional data - Flexible integration with DFT, CALPHAD, MD data	- Requires large, reliable training datasets - Limited interpretability (black-box models) - Predictions may lack physical rigor if not guided by theory	- Property prediction (hardness, strength, stability, Tc) - Materials discovery (composition optimization) - Surrogate models for phase diagrams and mechanical/thermal properties

**Table 2:** Comparison of processing routes for HEAs (casting, PM, AM, SPD, coatings) highlighting microstructure, benefits, and challenges.

Processing Route	Typical Microstructures	Benefits	Challenges
**Arc/Induction Melting + Casting**	- Dendritic structures - Segregation between dendritic core and interdendritic regions - Coarse grains	- Simple and widely used - Scalable to industrial levels - Produces bulk HEAs	- Segregation of elements - Porosity and shrinkage defects - Limited control over microstructure
**Powder Metallurgy (PM, including Hot Pressing/Spark Plasma Sintering)**	- Fine, equiaxed grains - Reduced segregation - Homogeneous distribution of phases	- Better compositional homogeneity - Lower processing temperatures - Near-net-shape fabrication possible	- Powder handling issues (oxidation, contamination) - Cost of powders - Limited scalability
**Mechanical Alloying (High-Energy Ball Milling) + Consolidation**	- Nanocrystalline or amorphous structures - Supersaturated solid solutions - Fine dispersion of secondary phases	- Ability to produce metastable/nanostructured HEAs - Extended solid solubility - High defect density enhances diffusion	- Contamination from milling media - Time and energy intensive - Requires subsequent consolidation (e.g., SPS, HIP)
**Additive Manufacturing (Selective Laser Melting, Electron Beam Melting, DED)**	- Fine columnar grains - Cellular dendritic substructures - Texture development	- High cooling rates → refined microstructures - Complex geometries possible - Tailored local properties via parameter control	- Residual stresses and cracking - Compositional inhomogeneity due to evaporation/segregation - Anisotropy in properties
**Thermomechanical Processing (Forging, Rolling, Extrusion)**	- Refined and recrystallized grains - Controlled texture - Secondary phase precipitation	- Improved mechanical properties (strength–ductility balance) - Tailorable microstructure - Industrially mature methods	- Requires high-temperature stability of HEAs - Equipment wear due to hardness of alloys - Multi-step processing
**Thin Film Deposition (Sputtering, Evaporation, CVD)**	- Nanocrystalline or amorphous films - Layered or single-phase structures depending on deposition parameters	- High compositional control - Ability to stabilize metastable phases - Functional coatings (hardness, corrosion, wear resistance)	- Limited to thin films - Expensive equipment - Scale-up challenges

**Table 3:** Comparison of the mechanical, chemical and functional properties of indicative HEAs.

Composition	Structure/Phase	Mechanical Properties				Chemical Properties	Functional Properties	Reference
		Yield Strength YS (MPa)	Ultimate Tensile/Compressive Strength UTS (MPa)	Hardness (HV or GPa)	Young’s Modulus (GPa)	Corrosion/Oxidation/Chemical Behavior		
**CrCoNi** (equiatomic, medium-entropy)	Bimodal microstructure with hard, non-recrystallized grains and soft, recrystallized grains after post-deformation heat treatments	1213 ± 13 (298 K)	1286 ± 24 (298 K)	—	—	Good room-temp corrosion similar to austenitic steels (depends on minor O/Cr oxides)	Exceptional strength-toughness; cryogenic strengthening (YS ↑ to 1556 ± 31 MPa at 77 K).	[192]
**CrMnFeCoNi** (Cantor alloy) (equiatomic)	FCC	~200–450 (typical room-T reported ranges)	~600–1000 (depends on processing)	~150–300 HV (varies)	~190–210 (typical FCC HEA)	Good corrosion resistance in many environments; oxide formation and localized corrosion depend on Cr/Ni content	Excellent cryogenic ductility/twinning-induced strengthening; widely used baseline HEA.	[193]
**Al_x_CoCrFeNi** (x = 0.1, 0.5, 1)	FCC (Al_0.1_) FCC + BCC (Al_0.5_) Mixed FCC + B2/ordered σ phase (Al_1_)	527.4 (YS)	943.3 (UTS)	507 HV (Al_1_) 275 HV (Al_0.5_) 142 HV (Al_0.1_)	—	Al addition alters high-temperature water vapor corrosion behavior. Al_3_Fe_5_O_12_, CoCr_2_O_4_ and NiCr_2_O_4_ oxides were formed in the Al_0.1_ and Al_0.5_ alloys. No oxides in the Al_1_	-	[194]
**NbMoTaW** (equiatomic refractory HEA)	BCC	— (RHEA brittle at RT in tension in some tests)	RHEA: σy ≈ 400 MPa at temperatures up to 1600 °C (high-T retention).	Very high hardness (GB/film values reported)	High E (refractory)	Excellent high-temperature oxidation resistance when alloyed appropriately; but oxidation can occur—coatings often required	Outstanding high-temperature strength retention; limited RT ductility in bulk.	[195]
**Ti_0.75_NbMoTaW** (Ti_x_NbMoTaW family)	BCC	YS = 1551 MPa (compressive, cast sample, RT)	Fracture (σf) ≈ 1856 MPa	—	273.78 GPa	Ti addition reduces VEC; improves ductility/toughness vs. NbMoTaW	-	[196]
**AlNbTiVCr** (lightweight HEA family)		YS per reports: several hundreds to >1000 MPa (processing dependent)	High specific strength (UTS/density)	Hardness 400–900 HV reported across LWHEA family	—	Designed to have lower density with retained strength; corrosion/oxidation depends on Al/Cr presence	Emphasis on high strength-to-weight for aerospace/automotive.	[197]
**AlCoCrFeNi_2.1_** (eutectic HEA, SLM/SPS)	FCC (Co, Cr, and Fe rich) and BCC (Al, Ni rich)	YS = 1437 ± 26 MPa	UTS = 1562 ± 33 MPa; elongation = 14 ± 1%	—	—	Eutectic microstructure changes corrosion/tribology	Eutectic HEAs can combine high strength and good ductility; suitable for wear applications.	[198]
